# Prevention and treatment of acute radiation-induced skin reactions: a systematic review and meta-analysis of randomized controlled trials

**DOI:** 10.1186/1471-2407-14-53

**Published:** 2014-01-31

**Authors:** Raymond Javan Chan, Joan Webster, Bryan Chung, Louise Marquart, Muhtashimuddin Ahmed, Stuart Garantziotis

**Affiliations:** 1 Cancer Care Services, Royal Brisbane and Women’s Hospital, Butterfield Street, Herston Q4029, Australia; 2 School of Nursing, Queensland University of Technology, Kelvin Grove Q4059, Australia; 3 Centre for Clinical Nursing, Royal Brisbane and Women’s Hospital, Butterfield Street, Herston Q4029, Australia; 4 Centre for Health Practice Innovation, Griffith University, Nathan Q4111, Australia; 5 Division of Plastic Surgery, QEII Health Science Centre, Halifax, Canada; 6 Statistics Unit, QIMR Berghofer Medical Research Institute, Herston, Brisbane Q4029, Australia; 7 Safety and Quality Unit, Royal Brisbane and Women’s Hospital, Butterfield Street, Herston Q4029, Australia

**Keywords:** Radiation induced skin reactions, Radiation dermatitis, Systematic review, Meta-analysis, Randomized controlled trials

## Abstract

**Background:**

Radiation-induced skin reaction (RISR) is a common side effect that affects the majority of cancer patients receiving radiation treatment. RISR is often characterised by swelling, redness, pigmentation, fibrosis, and ulceration, pain, warmth, burning, and itching of the skin. The aim of this systematic review was to assess the effects of interventions which aim to prevent or manage RISR in people with cancer.

**Methods:**

We searched the following databases up to November 2012: Cochrane Skin Group Specialised Register, CENTRAL (2012, Issue 11), MEDLINE (from 1946), EMBASE (from 1974), PsycINFO (from 1806), CINAHL (from 1981) and LILACS (from 1982). Randomized controlled trials evaluating interventions for preventing or managing RISR in cancer patients were included. The primary outcomes were development of RISR, and levels of RISR and symptom severity. Secondary outcomes were time taken to develop erythema or dry desquamation; quality of life; time taken to heal, a number of skin reaction and symptom severity measures; cost, participant satisfaction; ease of use and adverse effects. Where appropriate, we pooled results of randomized controlled trials using mean differences (MD) or odd ratios (OR) with 95% confidence intervals (CI).

**Results:**

Forty-seven studies were included in this review. These evaluated six types of interventions (oral systemic medications; skin care practices; steroidal topical therapies; non-steroidal topical therapies; dressings and other). Findings from two meta-analyses demonstrated significant benefits of oral Wobe-Mugos E for preventing RISR (OR 0.13 (95% CI 0.05 to 0.38)) and limiting the maximal level of RISR (MD -0.92 (95% CI -1.36 to -0.48)). Another meta-analysis reported that wearing deodorant does not influence the development of RISR (OR 0.80 (95% CI 0.47 to 1.37)).

**Conclusions:**

Despite the high number of trials in this area, there is limited good, comparative research that provides definitive results suggesting the effectiveness of any single intervention for reducing RISR. More research is required to demonstrate the usefulness of a wide range of products that are being used for reducing RISR. Future efforts for reducing RISR severity should focus on promising interventions, such as Wobe-Mugos E and oral zinc.

## Background

Radiation treatment remains an essential treatment for people with cancer and is associated with a number of short-term and long-term side-effects [1,2]. One of these side-effects is radiation-induced skin reaction (RISR), affecting up to 95% of people receiving radiation treatment for their cancer [3]. The reactions are a result of radiation treatment disrupting the normal process of cell division and regeneration, resulting in cell damage or cell death [4]. The damage can be a result of several processes, including a reduction of endothelial cell changes, inflammation, and epidermal cell death [5]. Radiation-induced skin reactions are often characterised by swelling, redness, pigmentation, fibrosis, and ulceration of the skin. Signs and symptoms are expressed as pain, warmth, burning, and itching of the skin [6]. The development of RISR may occur two to three weeks after treatment commences, and may persist up to four weeks after the treatment ends [7]. The factors influencing the development or severity of RISR have been classified in the literature as either being intrinsic or extrinsic [8]. Intrinsic factors include age, general health, ethnic origin, coexisting diseases, UV exposure, hormonal status, tumour site [8], and genetic factors [9]. Extrinsic factors include the dose, volume and fraction of radiation, radio-sensitisers, concurrent chemotherapy, and the site of treatment. These factors can be more broadly categorised into radiation treatment-related, genetic, and personal factors [3].

Interventions can be generally viewed as either preventive or management strategies [10]. Preventive strategies may include minimising irritants or irritations to the irradiated skin such as those associated with particular hygiene regimens, minimising friction, reducing the frequency of washing, avoiding the use of soap, cream and deodorants, and avoiding sun exposure [4,11]. Management strategies for established reactions may include active management of any reddening of the skin (erythema), any dry or moist shedding of the skin (desquamation), and ulceration of the skin, with topical preparations and dressings [8,10]. Erythema is defined as the redness caused by flushing of the skin due to dilatation of the blood capillaries in the dermis [12]. Dry desquamation is the shedding of the outer layers of the skin and moist desquamation occurs when the skin thins and then begins to weep as a result of loss of integrity of the epithelial barrier and a decrease in pressure exerted by plasma proteins on the capillary wall [13].

Radiation-induced skin reactions have an impact on the level of pain/discomfort experienced and the quality of life of those who undergo radiation treatment [2], and may even require changes to the person’s radiation schedule (if severe) [14]. In some cases, complex surgical reconstruction of damaged skin may be required [15]. Therefore, managing skin reactions is an important priority in caring for those who undergo radiation treatment [6]. Presently, a number of inconsistencies exist across radiation treatment centres globally with regard to the practice and recommendations given by health professionals to both prevent and manage this often painful side-effect of radiation treatment [8,10,16].

Efforts to guide practice in this area have led to the publication of seven systematic reviews [17-23]. We recently overviewed this literature and found conflicting conclusions and recommendations for practice [24,25]. There were also a number of methodological issues in many of reviews that we appraised, including the lack of duplicate assessment of study eligibility, inclusion of studies other than RCTs, lack of publication bias assessment, lack of declaration of conflict of interest, and inappropriate use of meta-analysis [24,25]. Consequently, we believed there was still a need for a high quality systematic review of interventions to prevent and manage RISR. Therefore, the aim of this systematic review was to assess the effects of interventions for preventing and managing RISR in people with cancer.

## Methods

### Inclusion criteria

All randomized controlled trials (RCT) providing a comparison between intervention types or a comparison between intervention and no intervention (usual care group) were considered. Participants were those receiving external beam radiation treatments. There were no restrictions on age of the participants, gender, diagnosis, previous or concurrent therapies, health status, dosage of treatment, location of irradiated area, or the setting where they received their radiation treatment. Studies which compared an intervention with the aim of preventing or managing RISRs were eligible. The inclusiveness of definitions of participants and interventions ensured that this review would be of use both to those in clinical practice as well as the wider population. Trials reporting the outcomes of interest listed below were included.

### Data sources, searches and study selection

We aimed to identify all relevant RCTs regardless of language or publication status (published, unpublished, in press, and in progress). We searched the following electronic databases: The Cochrane Skin Group Specialised Register, The Cochrane Central Register of Controlled Trials (CENTRAL) on The Cochrane Library (Issue 11, 2012), MEDLINE Ovid (1946 to 14/11/2012), EMBASE Ovid (1974 to 14/11/2012), PsycINFO Ovid (1806 to 14/11/2012), CINAHL EBSCO (1982 to 14/11/2012), and LILACS (1982 to 14/11/2012) (Please see Additional file 1). With reference to Additional file 1, we also searched trial registers, reference lists reported in relevant reviews and studies which were not identified via electronic searches, contents pages of selected journals (from inception to November 2012) for articles about interventions that aim to prevent or manage RISRs, abstracts from relevant conference proceedings, as well as the ProQuest Dissertations and Theses database.

Two review authors (RC and JW) independently pre-screened all search results (titles and abstracts) for possible inclusion based on the inclusion criteria, and those selected by either or both authors were subject to full-text assessment. These same two review authors independently assessed the selected articles against the inclusion criteria, and resolved any discrepancies by consensus. In this process, no arbiter was required. Studies that were excluded after full-text assessment are listed in Additional file 2, giving reasons for exclusion.

### Outcomes (Primary and Secondary)

Outcomes were classified as being related to either primary or secondary, and prevention or treatment (Table 1). We defined the primary outcome measures in this review as the development of RISR (Yes/No); the level of skin toxicity/reactions at one week and two weeks following the onset of the skin reaction; and the level of symptom severity at one week and two weeks following the onset of the skin reaction. During the review, we adjusted the time points of the secondary outcomes established in our review protocol. This was due to the difficulty of measuring the commencement of skin reactions of every trial participants. These outcome measures at the pre-specified time points were too restrictive and possibly unrealistic. These additional time points allow the inclusion of the average grading of RISR/ other symptom severity at certain weeks following the commencement of radiation treatment (e.g. maximum RISR throughout the treatment, at five or six weeks of radiation treatment, at completion of radiation treatment (usually between five to seven weeks), or at the last follow-up appointment (usually two weeks or four weeks after the completion of radiation treatment)). The maximum level of RISR represents the worst reaction associated with a given intervention or no intervention at all. These commonly reported outcome measures at these specific time points are considered clinically important, as the radiation dose reaches its highest accumulative level at the completion of radiation treatment.

**Table 1:** Primary and secondary outcomes of the review

**Outcome classification**	**Outcome description**
**Primary**	**Prevention**
	The development of a radiation-induced skin reaction (yes/no).
	**Treatment**
	Level of skin toxicity/reactions at one week and two weeks following the onset of the skin reaction.
	Level of symptom severity at one week and two weeks following the onset of the skin reaction (physical or psychological).
**Secondary**	**Prevention**
	Time taken to develop an erythema or dry desquamation.
	**Treatment**
	Quality of life.
	Time taken to heal.
	Level of skin toxicity/reactions at the completion of treatment and at the last follow-up.
	Maximum level of skin toxicity/reactions reported.
	Level of symptom severity at any time following the onset of treatment (physical or psychological), at the completion of radiation treatment and at the last follow-up.*
	Maximum level of symptom severity (physical or psychological) reported.*
	**Prevention and Treatment**
	Cost of the interventions (both direct and indirect cost, both to the participant and the health system).
	Participant satisfaction.
	Ease of use.
	Adverse effects (including allergic reactions).

*All symptoms reported by eligible trials were included.

### Data extraction

A data extraction form (see Additional file 3) was developed, piloted (with three studies) and amended. Two individuals, with at least one being RC or JW, independently extracted data using the data extraction form for each study. Any errors or inconsistencies were resolved after consulting the original source and consensus. RC entered the data into RevMan 5, with JW and LM checking the accuracy of all data entry.

### Risk of bias assessment

We assessed and reported on the risk of bias of included studies in accordance with the guidelines in the *Cochrane Handbook for Systematic Reviews of Interventions*[26], which recommends the explicit reporting of individual domains including sequence generation, allocation concealment, blinding of participants, personnel and outcome assessors, incomplete outcome data, selective reporting, and other sources of bias. Two review authors independently assessed the risk of bias in included studies, with any disagreements resolved by discussion and consensus. This led to an overall assessment of the risk of bias of the included studies [27]. We assessed each of the risk of bias items as low, high, or unclear.

### Data synthesis and analysis

We analysed data using The Cochrane Collaboration’s Review Manager 5 (RevMan 5). We examined the data from included studies for descriptive synthesis and pooled data where trials were sufficiently homogeneous in design, methodology, and outcomes. At the protocol stage, it was expected that interventions might be classified as either preventative or management strategies [10], and that the interventions could be used for either or both purposes [17]. We have reported the data according to the definitions of included outcomes (whether preventative or management in nature) in this review. We considered studies with less than 100 participants small, studies with between 101 to 200 participants medium and studies with more than 201 participants large.

If included studies were sufficiently similar in terms of population, inclusion criteria, interventions and/or outcomes (including the time(s) at which these were assessed throughout the radiation regimen), we considered pooling the data statistically using meta-analysis. These were reported as pooled mean differences (MD) or Standardised mean differences (SMD) (continuous variables), or odds ratios (OR) (dichotomous variables) and corresponding 95% confidence interval (CI). Numbers needed to treat (NNT) for benefit or harm was not calculated due to the low number of meta-analysis conducted in this review. For survival data, we used hazard ratios and corresponding 95% CI for comparison. If any data was not quoted in studies, then we requested these from authors. Alternatively, we attempted to calculate this from available summary statistics (observed events, expected events, variance, confidence intervals, P values, or survival curves) according to the methods proposed by Parmer and colleagues [28]. However, this was not always possible due to the lack of data provided in the paper/by authors despite attempts to contact them for this information.

Heterogeneity was tested using the I^2^ statistic (which was used to describe the percentage of the variability in effect estimates that was due to heterogeneity rather than sampling error). A value greater than 50% was considered to represent substantial heterogeneity [26], and we explored heterogeneity and possible reasons. We undertook random-effects analyses if I^2^ was greater than 50%. With regards to the assessment of publication bias, it is recommended that a funnel plot should only be constructed when there are at least 10 studies in a meta-analysis [26]. Therefore, we did not construct a funnel plot to assess the possibility of publication bias because there were too few trials per meta-analysis (all <3).

## Results

### Study selection

**Figure 1:**
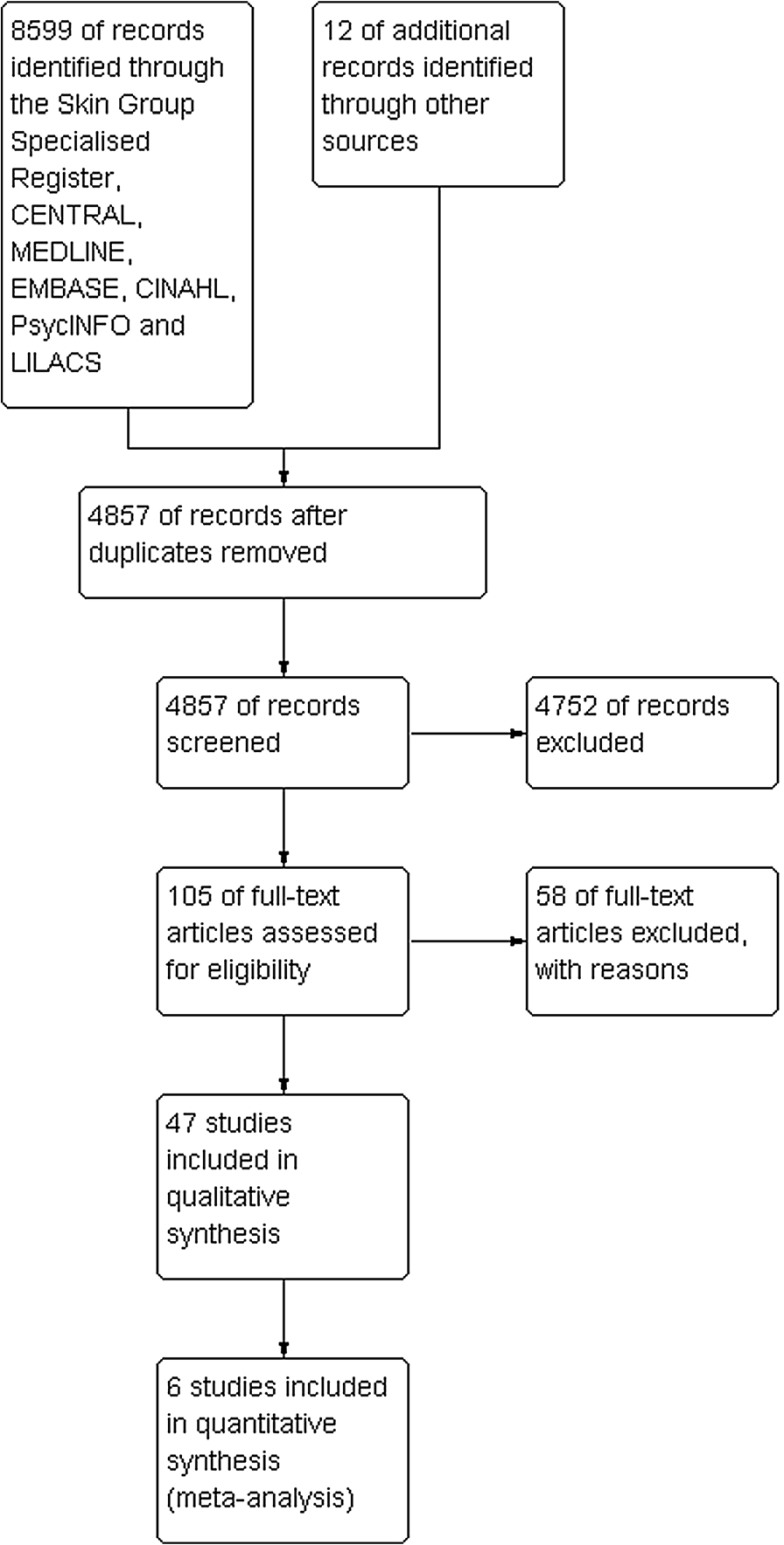
Study flow diagram.

**Figure 2:**
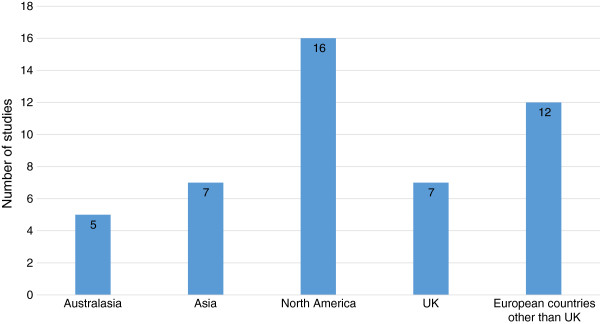
Number of included studies by country or region.

**Figure 3:**
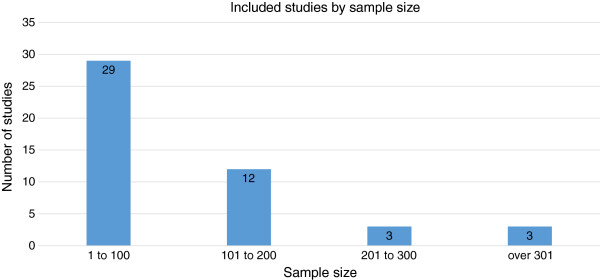
Number of included studies sample size.

The different steps of the electronic search are illustrated in Figure 1. In total, we identified 4857 citations from the electronic database searches after removing the duplicates. After we screened all the titles and abstracts, 105 articles were potentially relevant and we retrieved them in full text. Of these 105 titles, we included 47 studies involving 5688 participants and excluded 58 which did not meet the selection criteria. The characteristics of all included studies are included in Additional file 4. Classification of included studies by countries or regions is illustrated in Figure 2, with the majority of studies coming from North America (n = 16). In addition, sample size variation between trials is illustrated in Figure 3. The majority (n = 29) of studies included fewer than 100 patients (small). Twelve trials included 101–200 participants (medium), and six trials included over 201 participants (large), with a maximum number of participants being 547. All studies were undertaken with adult patients except for one that included patients between three and 21 years of age [29]. Of the 47 included studies, six trials investigated the effects of oral systemic therapies [30-35]; two examined washing practices [36,37], four examined deodorant/antiperspirant use [38-41]; five examined steroidal topical therapies [42-46]; 23 examined non-steroidal topical therapies [11,29,47-67]; six examined dressing interventions [68-73]; and one examined light emitting diode photo-modulation [74].

### Risk of bias assessment

**Figure 4:**
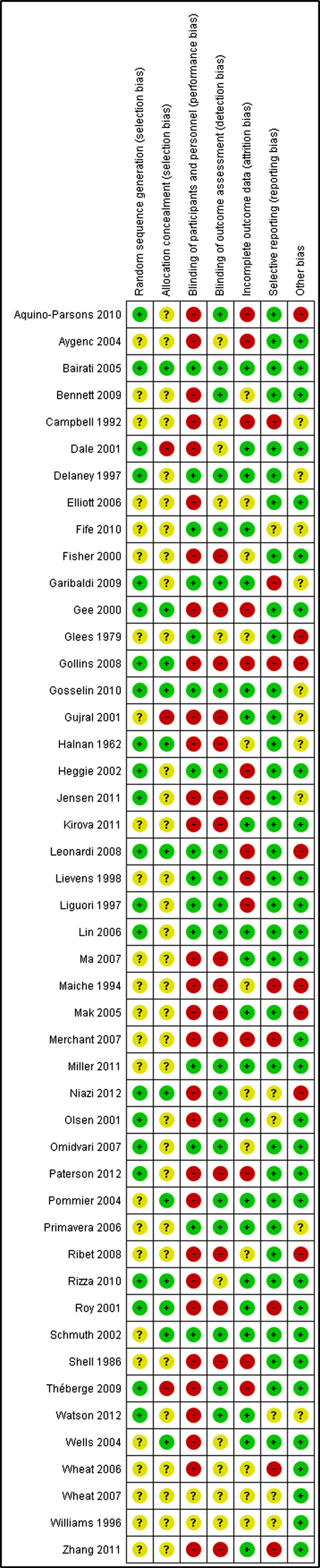
Risk of bias summary: review authors’ judgements about each risk of bias item for each included study.

Risk of bias assessment is reported in Figure 4. Thirty-six studies were considered high risk of bias (plausible bias that seriously weakens confidence in the results), because one or more domains received a judgement of high risk [11,29,30,32-34,36-43,48-50,52-60,62-64],[67-73]. Ten studies were rated as unclear risk of bias (plausible bias that raises some doubt about the result) because one or more criteria were assessed as unclear [35,44-47,51,61,65,66,74]. One study [31] was considered low risk of bias, because all domains received a judgement of low risk (see Figure 4).

With regards to allocation concealment (selection bias), the method used to generate the allocation sequence was clearly described in 22 studies [31,32,35,37,39-41,43,45,47],[50-53,55,56,59,63,68,69,71],[72], but not in the 25 remaining studies. Of the 47 studies, 22 studies adequately reported allocation concealment [31,32,35,37,39-41,43,45,47],[50-53,55,56,59,63,68,69,71],[72]. The method used to conceal the allocation sequence was not reported in the remaining studies, thus receiving a judgement of unclear risk of bias for this domain.

Performance and detection bias, blinding for participants and personnel from knowledge of which intervention a participant received was achieved in a number of placebo trials. All other open label trials and trials that compared a single intervention and usual care/institutional preferences received a judgement of high risk for this domain. Of the 47 studies, 16 studies described in sufficient detail how blinding of participants and personnel was achieved [31,34,35,42,44-47,50-52,55,56],[58,59,74]. Blinding for outcome assessors was sufficiently described in 21 studies [31,34,35,38,40,41,44-47,50-52],[55,56,59-61,68,71,74]. The remaining studies either did not have adequate reporting of how blinding was achieved or did not blind the participants, personnel and/or outcome assessors at all.

With regards to attrition bias, incomplete outcome data appears to have been adequately addressed in 21 studies [11,31-33,35,37,41,44,46,47],[50,51,54,57,59-61,63,67,70],[74]. Outcomes were reasonably well-balanced across intervention groups/control groups, with similar reasons for missing data across the groups and intention-to-treat analysis conducted. However, in 14 studies, there were concerns about unbalanced groups with missing data or the lack of intention-to-treat analysis [29,30,34,36,39,40,52,53],[55,56,68,69,72,73]. The remaining 12 studies did not provide sufficient information to allow for a clear judgement of the risk of bias for this domain.

In terms of selective reporting (reporting bias), the protocols were not available for any of the included studies. Based on the information in the methods section of the reports, 33 studies appear to have reported all pre-specified outcomes and were therefore judged to be free of selecting reporting [11,30-35,38-40,42-49,51-57,60-63,68,70],[72,73]. The remaining 14 studies were judged to be unclear or high bias. In the judgement of risk of bias for this domain, we also took into consideration whether trials reported on some of the important and expected outcomes such as RISR severity, pain and itch. Studies that did not report these outcomes received a judgement of unclear risk of bias.

For other potential sources of bias, we judged as unclear or high risk of bias in 18 studies (e.g. declarations of potential conflicts of interest or funding support were frequently unreported, or the report did not clearly state to what extent any support might have posed a risk of bias) [33,36,41-43,47,50,51,53,55],[58,61,62,68-71,74]. The included studies received a low risk of bias if no other potential threats to validity were identified.

### Data synthesis

Table 2 outlines the results of all analyses carried out for the purpose of this review. However, results of particular interest pertaining to both prevention and treatment interventions are summarized below.

**Table 2:** Summary of results

**Comparison of interventions**	**Included studies, sample size and treatment areas**	**Outcome type**	**Outcome**	**Results/effect size**
**1. Oral systemic medications**				
1.1 Oral Wobe-Mugos versus no medication	Dale, 2001; Gujral, 2001 N = 219	Prevention	**Primary**	
			Development of RISR (Yes/No) (Dale, 2001 & Gujral, 2001)	Meta-analysis:
				OR 0.13, 95% CI 0.05 to 0.38, p < 0.0005
				(Favouring Oral Wobe-Mugos)
		Treatment	**Secondary**	
			Maximum Levels of RISR (RTOG/EORTC criteria, with a possible range of 0–4) (Dale, 2001 & Gujral, 2001)	Meta-analysis:
				MD -0.92, 95% CI -1.36 to -0.48, p < 0.0001
				(Favouring Oral Wobe-Mugos)
1.2 Oral Pentoxifylline versus no medication	Aygenc, 2004	Prevention	**Primary**	
	N = 78		Development of RISR (Yes/No)	OR 0.18, 95% CI 0.01 to 3.95, p = 0.28
		Treatment	**Secondary:**	
			Adverse Effects (Yes/No)	OR 17.24, 95% CI 0.95 to 313.28, p = 0.05
1.3 Oral Antioxidant versus placebo	Bairati, 2005	Treatment	**Secondary:**	MD -0.06, 95% CI -0.15 to 0.03, p = 0.17
	N = 545		RISR at the end of radiation treatment (RTOG criteria, with a possible range of 0–4)	
			RISR at four weeks after the end of radiation treatment (RTOG criteria, with a possible range of 0–4)	MD 0.00, 95% CI -0.08 to 0.08, p = 1.00
			Global quality of life at the end of radiation treatment (QoLC30, with a possible range of 0–100)	MD 0.00, 95% CI -3.95 to 3.95, p = 1.00
			Global quality of life at four weeks after the end of radiation treatment (QoLC30, with a possible range of 0–100)	MD -2.00, 95% CI -5.29 to 1.29, p = 0.23
			Skin-related quality of life at the end of radiation treatment (HNC-QoL, with a possible range of 0–7 with 7 representing better quality of life)	MD 0.10, 95% CI -0.16 to 0.36. p = 0.46
			Skin-related quality of life at weeks after the end of radiation treatment (HNC-QoL, with a possible range of 0–7 with 7 representing better quality of life)	MD 0.00, 95% CI -0.12 to 0.12, p = 1.00
1.4 Oral sucralfate suspension versus placebo	Lievens, 1998	Treatment	**Secondary:**	
	N = 83		Maximum levels of RISR (Scoring system developed by authors, with a possible range of 0–6)	MD 0.20, 95% CI -0.34 to 0.74, p = 0.47
			Adverse effect (measured as mean peak nausea, scoring system developed by authors, 0 = none, 4 = vomiting resistant to medication)	MD -0.22, 95% CI -0.61 to 0.17, p = 0.27
1.5 Oral zinc supplementation versus placebo	Lin, 2006	Treatment	**Secondary:**	
	N = 97		RISR at the completion of radiation treatment (RTOG criteria, with a possible range of 0–4)	MD -0.50, 95% CI -0.58 to -0.42, p < 0.00001
				(Favouring oral zinc supplementation)
**2. Skincare practices (washing practices and deodorant use)**				
2.1 Washing with soap versus no washing	Campbell, 1992; Roy, 2001	Prevention	**Primary:**	
			Development of RISR (Yes/No) (Roy, 2001)	OR 0.32, 95% CI 0.01 to 8.05, p = 0.49
	N = 167			
		Treatment	**Secondary:**	
			Itch at the end of treatment (week six) and the two-week follow-up (week eight) (EORTC/RTOG criteria, with a possible score of 0–3) (Campbell, 1992)	Week 6- MD -0.43, 95% CI -0.97 to 0.11, p = 0.12,Week 8- MD-0.40, 95% CI -0.81 to 0.01, p = 0.06 (Favouring washing with soap)
			Erythema at the end of treatment (week six) and the two-week follow-up (week eight) (EORTC/RTOG criteria, with a possible score of 0–3) (Campbell, 1992)	Week 6- MD-0.40 95% CI -0.77 to -0.03, p = 0.03, Week 8-MD -0.21, 95% CI -0.52 to 0.10, p = 0.18
			Desquamation at the end of treatment (week six) and the two-week follow-up (week eight) (EORTC/RTOG criteria, with a possible score of 0–3) (Campbell, 1992)	Week 6- MD -0.47, 95% CI -0.83 to -0.11, p = 0.01, Week 8- MD -0.82, 95% CI -1.16 to -0.48, p < 0.00001 (Favouring washing with soap)
2.2 Washing with water versus no washing	Campbell, 1992	Treatment	**Secondary:**	
	N = 58		Itch at the end of treatment (week six) and the two-week follow-up (week eight) (EORTC/RTOG criteria, with a possible score of 0–3)	Week 6- MD -0.27, 95% CI -0.83 to 0.29, p = 0.35, Week 8- MD -0.46, 95% CI -0.83 to -0.09, p = 0.01 (Favouring washing with water)
			Erythema at the end of treatment (week six) and the two-week follow-up (week eight) (EORTC/RTOG criteria, with a possible score of 0–3)	Week 6- MD -0.34, 95% CI -0.69 to 0.01, p = 0.06, Week 8- MD -0.44, 95% CI -0.72 to -0.16, p = 0.002 (Favouring washing with water)
			Desquamation at the end of treatment (week six) and the two-week follow-up (week eight) (EORTC/RTOG criteria, with a possible score of 0–3)	Week 6- MD -0.59, 95% CI -0.94 to -0.24, p = 0.001, Week 8- MD -0.62, 95% CI -0.96 to -0.28, p = 0.0004 (Favouring washing with water)
2.3 Washing with water versus washing with soap	Campbell, 1992	Treatment	**Secondary:**	
	N = 64		Itch at the end of treatment (week six) and the two-week follow-up (week eight) (EORTC/RTOG criteria, with a possible score of 0–3)	Week 6- MD 0.16, 95% CI -0.35 to 0.67, p = 0.54, Week 8- MD -0.06, 95% CI -0.39 to 0.27, p = 0.72
			Erythema at the end of treatment (week six) and the two-week follow-up (week eight) (EORTC/RTOG criteria, with a possible score of 0–3)	Week 6- MD 0.06, 95% CI -0.26 to 0.38, p = 0.71, Week 8- MD -0.44, 95% CI -0.72 to -0.16, p = 0.001 (Favouring washing with water)
			Desquamation at the end of treatment (week six) and the two-week follow-up (week eight) (EORTC/RTOG criteria, with a possible score of 0–3)	Week 6- MD -0.12, 95% CI -0.51 to 0.27, p = 0.54, Week 8- MD 0.20, 95% CI -0.16 to 0.56, p = 0.27
2.4 Deodorant versus no deodorant	Bennett, 2009; Gee, 2000; Theberge, 2009; Watson, 2012	Prevention	**Primary:**	
			Development of RISR (Yes/No) (Bennett, 2009 & Gee, 2000)	Meta-analysis:
				OR 0.80, 95% CI 0.47 to 1.37, p = 0.42
			Development of RISR in patients with axilla treated (Yes/No) (Bennett, 2009)	OR 0.06, 95% CI 0.01 to 0.60, p = 0.02
	N = 509	Treatment	**Secondary:**	
			RISR at the end of radiation treatment and at the two-week follow-up (CTCAE criteria version 3, with a possible range of 0–3) (Watson, 2012)	End of treatment- MD 0.01, 95% CI -0.17 to 0.19, p = 0.91, Two-week follow-up- MD 0.01, 95% CI -0.21 to 0.23, p = 0.93
			Maximum RISR rated by researcher (RTOG criteria, with a possible range of 0–3) (Bennett, 2009)	MD = -0.74, 95% CI -1.22 to -0.26, p = 0.003
				(Favouring deodorant)
			Moderate-to-severe pain at the end of radiation treatment and at the two-week follow-up (Yes/No) (Theberge, 2009)	End of treatment- OR 0.77, 95% CI 0.29 to 2.09, p = 0.61, Two-week follow-up- OR 2.16, 9% CI 0.65 to 7.14, p = 0.21
			Pruritus at the end of radiation treatment and at the two-week follow-up (Yes/No) (Theberge, 2009)	End of treatment- OR 2.62, 95% CI 1.01 to 6.78, p = 0.05, Two-week follow-up- OR 1.47, 95% CI 0.57 to 3.77, p = 0.42
			Sweating at the end of radiation treatment and at the two-week follow-up (Yes/No) (Theberge, 2009)	End of treatment- OR 0.34, 95% CI 0.12 to 0.93, p = 0.04, Two-week follow up- OR 0.70, 95% CI 0.25 to 1.99, p = 0.51
**3. Steroidal topical ointment/cream**				
3.1 Topical 0.1% mometasone furoate cream versus placebo	Miller, 2011	Prevention	**Primary:**	
	N = 166		Development of RISR (Yes/ No)	OR 0.60, 95% CI 0.28 to 1.31, p = 0.20
		Treatment	**Secondary:**	
			RISR at the two-week follow-up after the completion of radiation treatment (CTCAE criteria version 3.0, with a possible range of 0–3)	MD -0.39, 95% CI -0.80 to 0.02, p = 0.06
			Maximum RISR level (CTCAE criteria version 3.0, with a possible range of 0–3)	MD -0.10, 95% CI -0.35 to 0.15, p = 0.43
3.2 Topical betamethasone cream versus placebo	Omidvari, 2007	Prevention	**Primary:**	
	N = 36		Development of RISR (Yes/No)	There was an equal proportion of people developing a RISR (summary statistics not estimated)
		Treatment	**Secondary:**	
			RISR at the end of treatment (week five) and the two-week follow-up (week seven) (RTOG criteria, with a possible range of 0–4)	End of treatment- MD -0.10, 95% CI -0.28 to 0.08, p = 0.28, two-week follow-up- MD -0.55, 95% CI -0.71 to -0.39, p < 0.00001 (Favouring topical betamethasone cream)
			Maximum level of RISR (RTOG criteria, with a possible range of 0–4)	MD -1.62, 95% CI -2.03 to -1.21, p < 0.00001 (Favouring topical betamethasone cream)
3.3 Topical betamethasone versus no topical treatment	Omidvari, 2007	Prevention	**Primary:**	
	N = 36		Development of RISR (Yes/ No)	There was an equal proportion of people developing a RISR (summary statistics not estimated)
		Treatment	**Secondary:**	
			RISR at the end of treatment (week five) and two weeks after treatment (week seven) (RTOG criteria, with a possible range of 0–4)	End of treatment- MD -0.40, 95% CI -0.62 to -0.15, p = 0.002, two-week follow-up- MD -0.30, 95% CI -0.53 to -0.07, p = 0.01
				(Favouring topical betamethasone cream)
			Maximum level of RISR (RTOG criteria, with a possible range of 0–4)	MD -0.27, 95% CI -0.75 to 0.21, p = 0.27
3.4 Topical corticosteroid versus another topical corticosteroid	Glees, 1979	Prevention	**Primary:**	
	N = 53		Development of RISR (Yes/ No)	OR 3.35, 95% CI 0.13 to 86.03, p = 0.46
3.5 Topical corticosteroid plus antibiotics versus corticosteroid alone	Halnan, 1962	Prevention	**Primary:**	
	N = 20		Development of RISR (Yes/ No)	There was an equal proportion of people developing a RISR (summary statistics not estimated)
3.6 Topical corticosteroid plus antibiotics versus no treatment	Halnan, 1962	Prevention	**Primary:**	
	N = 20		Development of RISR (Yes/ No)	OR 0.07, 95% CI 0.01 to 0.84, p = 0.04
				(Favouring topical corticosteroid plus antibiotics)
3.7 Topical corticosteroid versus dexpanthenol	Schmuth, 2002	Treatment	**Secondary:**	
	N = 21		Levels of RISR at the end of radiation treatment (week six) (The clinical symptom score with a possible range of 0–3)	MD -0.10, 95% CI -0.57 to 0.37, p = 0.68
			Levels of RISR at the two-week follow-up after the end of radiation treatment (week eight) (The clinical symptom score with a possible range of 0–3)	MD -1.40, 95% CI -1.97 to -0.83, p < 0.00001
				(Favouring topical corticosteroid)
**4. Dressings**				
4.1 Hydrogel dressing versus Gentian violet dressing	Gollins, 2008	Treatment	**Secondary:**	
	N = 20		Time to heal (days)	HR 7.95, 95% CI 2.20-28.68, p = 0.002
				(Favouring hydrogel dressing)
			Adverse events (measured as stinging, yes or no)	OR 3.89, 95% CI 0.62 to 24.52, p = 0.15
4.2 Gentian violet dressing versus non-adherent dressing	Mak, 2005	Treatment	**Secondary:**	
	N = 39		Time to heal (days)	HR 0.73, 95% CI 0.52-1.03, p = 0.07
			Pain at week two after the application of dressing (Scoring system developed by authors, with a possible range of 0–5)	MD -0.29, 95% CI = -0.66 to 0.08, p = 0.13
4.3 Silver nylon dressing versus standard care	Niazi, 2012		RISR severity at the end of radiation treatment (CTCAE criteria version 4, with a possible range of 0–4)	MD = -0.86, 95% CI -1.59 to -0.13, p = 0.02
	N = 40			(Favouring silver nylon dressing)
4.4 MVP dressings versus Lanolin dressing	Shell, 1986	Shell 1986 compared moisture vapour permeable (MVP dressing) compared with lanolin dressings. We were unable to extract data from the study. Insufficient information was provided in relation to the time to healing outcome as well as the RISR scores (no SD provided). The trial authors reported “the trend to faster healing in the MVP group was not statistically significant”.		
	N = 16			
4.5 Mepilex lite dressing versus aqueous cream	Paterson, 2012	Paterson 2012 compared Mepilex lite dressing with aqueous cream alone. We were unable to extract data from the study. However, the trial authors reported that ”Mepilex Lite dressings did not significantly reduce the incidence of moist desquamation, but did reduce the overall severity of skin reactions by 41% (p < 0.001), and the average moist desquamation score by 49% (p = 0.043).“ The trial authors were contacted for further information. However, no replies were received at the time of publishing this review.		
	N = 74			
**5. Non-steroidal ointment/cream**				
5.1 Hyaluronic acid versus placebo cream	Kirova, 2011; Leonardi, 2008; Liguori, 1997; Primavera, 2006	Prevention	**Primary:**	
			Development of RISR (Yes/No) (Leonardi, 2008)	OR 0.39, 95% CI 0.01 to 10.10, p = 0.57
		Treatment	**Secondary:**	
			Severe pain (>2) at week one, week two and week three of radiation treatment (as defined as >2 on a visual analogue scale, Yes/No) (Kirova 2011)	Week One- OR 1.25, 95% CI 0.67 to 2.32, p = 0.49, Week Two- OR 1.79, 95% CI 0.97 to 3.27, p = 0.06, Week Three- OR 1.30, 95% CI 0.66 to 2.59, p = 0.45
	N = 384			
			Quality of life at week four of radiation treatment (EORTC CLC Q30) (Kirova 2011)	MD -0.10, 95% CI -6.75 to 6.55, p = 0.98
			RISR severity at the end of radiation treatment (Scoring system developed by authors, with a possible range of 0–6) (Liguori 1997)	MD -0.73, 95% CI -1.04 to -0.42, p < 0.00001
				(Favouring hyaluronic acid)
			RISR severity at four weeks after radiation treatment completion (Scoring system developed by authors, with a possible range of 0–6) (Liguori 1997)	MD -0.35, 95% CI -0.68 to -0.02, p = 0.04
				(Favouring hyaluronic acid)
			Maximum RISR over the duration of radiation treatment (CTCAE, with a possible range of 0–4) (Leonardi, 2008)	MD-0.95, 95% CI -1.23 to -0.67, p < 0.00001
				(Favouring hyaluronic acid)
			Pain, itching and burning at four weeks of radiation treatment (0–10 cm visual analogue scale, with a possible range of 0–10) (Leonardi, 2008)	Pain- MD -0.50, 95% CI -1.72 to 0.72, p = 0.42, Itching- MD -0.18, 95% CI -1.39 to 1.03, p = 0.77, Burning- MD -0.91, 95% CI -2.01 to 0.19, p = 0.10
			Adverse effects (yes/no) (Leonardi, 2008)	OR 0.17, 95% CI 0.02 to 1.65, p = 0.13
5.2 Aloe vera versus aqueous cream	Heggie, 2002	Heggie 2002 reported that “aqueous cream was significantly better than aloe vera in reducing dry desquamation and pain related to treatment”. However, this study did not contain data or summary statistics concerning the outcomes as defined by this review.		
	N = 225			
5.3 Aloe vera gel and soap versus soap alone	Olsen, 2001	Olsen 2001reported that “when the cumulative of radiation dose was high (>2700 cGy), the median time was give weeks prior to any skin changes in the aloe/soap arm versus three weeks in the soap only arm. When cumulative dose increases over time, there seems to be a protective effect of adding aloe to the soap regimen.” However, this study did not contain data or summary statistics concerning the outcomes as defined by this review.		
	N = 73			
5.4 Aloe vera gel versus placebo	Williams, 1996	Williams 1996 reported that “skin dermatitis scores were virtually identical on both treatment arms, and that, aloe vera gel does not protect against radiation treatment-induced dermatitis”. However, this study did not contain data or summary statistics concerning the outcomes as defined by this review.		
	N = 194			
5.5 Aloe vera gel versus an Anionic Phospholipid-Based (APP) cream	Merchant, 2007	Merchant 2007 (n = 194) reported that statistically significant differences were found favouring the APP cream over the aloe vera gel in a number of outcomes including skin comfort, RISR skin severity. However, this study did not contain data or summary statistics concerning the outcomes as defined by this review.		
	N = 194			
5.6 Trolamine versus usual care as per institutional preference	Elliott, 2006; Fisher, 2000	Prevention	**Primary:**	
			Development of RISR (yes/no) (Elliot 2006)	OR 0.40, 95% CI 0.08 to 2.11, p = 0.28
	N = 462	Treatment	**Secondary:**	
			Maximum levels of RISR (NCI CTC criteria and RTOG criteria, with a possible range of 0–4) (Eilott, 2006 & Fisher,2000)	Meta-analysis:
				MD 0.00, 95% CI -0.13 to 0.13, p = 0.97
5.7 Trolamine versus Placebo	Gosselin, 2010		Patient satisfaction (Scoring system developed by authors, with a possible range of 0–5, 5-best satisfaction)	MD 1.12, 95% CI 0.56 to 1.68, p < 0.00001 (Favouring trolamine)
	N = 102			
			Ease of use (Scoring system developed by authors, with a possible range of 0–5, 5-highest level of ease)	MD 0.44, 95% CI 0.01 to 0.87, p = 0.04
				(Favouring trolamine)
5.8 Trolamine versus Calendula	Pommier, 2004	Treatment	**Secondary:**	OR 7.68, 95% CI 3.07 to 19.17, p < 0.0001
	N = 254		Ease of use (measured as difficult to use- yes or no)	(Favouring trolamine)
			Allergic reaction (yes/no)	OR 0.11, 95% CI 0.01 to 2.05, p = 0.14
5.9 Trolamine versus ETA Gel (99% Avene Thermal Spring Water)	Ribet, 2008	Treatment	Secondary:	
	N = 54		RISR severity at the end of radiation treatment (NCI CTC criteria, with a possible range of 0–4)	MD -0.14, 95% CI -0.58 to 0.30, p = 0.53
5.10 Trolamine versus topical Qingdiyou medication	Zhang, 2011	Zhang 2011 compared trolamine and topical qingdiyou medication. We could not extract data from this study. The trial authors reported that patients who received qingdiyou medication had significantly less severe RISR (p < 0.05). The trial authors did not provide a time point as to when the assessments were undertaken. We attempted contacted authors for further information. However, no further information was available.		
	N = 72			
5.11 Sorbolene versus wheatgrass extract cream	Wheat, 2006; Wheat, 2007	Wheat 2006 (n = 30) and Wheat 2007 (n = 20) compared Sorbolene and wheatgrass extract cream. We could not extract data from these two studies. The trial authors did not provide SE, SD or 95% CI for the mean scores reported. The trial authors were contacted for further information. However, no replies were received at the time of publishing this review. Both studies reported that there were no statistically significant differences between the two arms with respect to the peak RISR severity or time to peak RISR rating. The trial authors reported a statistically significant improvement in quality of life of patients in the wheatgrass group at week five and week six of radiation treatment.		
	N = 50			
5.12 Sucralfate mixed with Sobolene (10% w/w (50 g of sucralfate crushed in 500 g of sorbolene) versus Sorbolene cream	Delaney, 1997	The trial authors of Delaney 1997 reported that mean time to healing for the sucralfate and control groups, respectively were 14.8 days (coefficient of variation (c.v. = 70%) and 14.2 days (c.v. = 75%). The ratio of mean times to healing was 1.043 and was not statistically different from 1. (p = 0.86, 95% CI 0.65, 1.67). Estimates of the SD could not be calculated as it was unsure whether the c.v. data presented by the authors was based on the log transformed time-to-heal data or the untransformed data. The trial authors reported that “there was no statistically significant difference was found between the two arms in either from randomisation to healing or improvement in pain score”. We could not extract data from this study. The trial authors were contacted for further information. However, no replies were received at the time of publishing this review.		
	N = 39			
5.13 Sucralfate cream versus placebo cream	Maiche, 1994	Maiche 1994 compared sucralfate cream and placebo cream. The trial authors reported that “grade 1 and grade 2 reactions appeared significantly later on the areas treated with sucralfate cream. Grade 2 reactions were observed highly significantly more often at four weeks (p = 0.01) and at five weeks (p < 0.05) in favour of sucralfate. No allergic reactions were observed in either group. No other data were available after attempts to contact trial authors for more information.		
	N = 44			
5.14 Sucralfate cream versus aqueous cream	Wells, 2004	Wells 2004 compared Sorbolene and aqueous cream. We could not extract data from this study. The trial authors did not provide SE, SD or 95% CI for the mean scores reported. However, the authors reported that no statistically significant differences were found in the severity of skin reactions suffered by patients in either of the treatment arms.		
	N = 357			
5.15 Non-steroidal restitutio restructuring cream formula A and non-steroidal restitutio restructuring cream formula B	Garibaldi, 2009	Prevention	**Primary:**	
	N = 64		Development of RISR (yes/no)	OR 0.64 95% CI 0.22 to 1.88, p = 0.41
5.16 WO1932 oil in water emulsion versus usual care/untreated	Jenson, 2011	Treatment	**Secondary:**	
	N = 66		RISR severity at the end of radiation treatment (Oncology Nursing Society Skin Reaction Scoring System, with a possible range of 0–3)	MD -0.21, 95% CI -0.43 to 0.01, p = 0.07
5.17 Aquaphor ointment versus placebo	Gosselin, 2010	Treatment	**Secondary:**	
	N = 106		Patient satisfaction (Scoring system developed by authors, with a possible range of 0–5, 5-best satisfaction)	MD 0.59, 95% CI 0.04 to 1.15, p = 0.04
				(Favouring aquaphor ointment)
			Ease of use (Scoring system developed by authors, with a possible range of 0–5, 5-best level of ease)	MD -0.10, 95% CI -0.61 to 0.41, p = 0.70
5.18 RadiaCare gel versus placebo	Gosselin, 2010	Treatment	**Secondary:**	
	N = 106		Patient satisfaction (Scoring system developed by authors, with a possible range of 0–5, 5-best satisfaction)	MD 0.91, 95% CI 0.36 to 1.46, p = 0.001
				(Favouring radiacare gel)
			Ease of use (Scoring system developed by authors, with a possible range of 0–5, 5-best level of ease)	MD 0.16, 95% CI -0.30 to 0.62, p = 0.49
5.19 Topical Lian Bai liquid versus no Lian Bai liquid	Ma, 2007	Prevention	Development of RISR (yes or no)	OR 0.04, 95% CI 0.01 to 0.12, p < 0.00001
	N = 126			(Favouring topical lian bai liquid)
5.20 Formulation A topical cream (capprais spinosa, opuntia coccinellifera and olive leaf extract) versus no treatment	Rizza, 2010	Treatment	**Secondary:**	
	N = 44		Maximum RISR over eight weeks (modified RTOG criteria, with a possible range of 0–4)	MD -1.17, 95% CI -1.59 to -0.75, p < 0.00001
				(Favouring formulation A topical cream)
5.21 Formulation B topical cream (non-steroidal water based emulsion) versus no Treatment	Rizza, 2010	Treatment	**Secondary:**	
	N = 42		Maximum RISR over eight weeks (modified RTOG criteria, with a possible range of 0–4)	MD -0.79, 95% CI -1.21 to -0.37, p < 0.00001
				(Favouring formulation B topical cream)
5.22 Formulation A topical cream (capprais spinosa, opuntia coccinellifera and olive leaf extract) versus formulation B topical cream (non-steroidal water based emulsion)	Rizza, 2010	Treatment	**Secondary:**	
	N = 50		Maximum RISR over eight weeks (modified RTOG criteria, with a possible range of 0–4)	MD -0.38, 95% CI -0.69 to -0.07, p = 0.02
				(Favouring formulation A topical cream)
**6. Other interventions**				
6.1 LED versus no LED treatment	Fife, 2010	Prevention	**Primary:**	
	N = 33		Development of RISR	OR 3.83, 95% CI 0.14 to 101.07, p = 0.42
		Treatment	Secondary:	
			Levels of RISR at week five (CTCAE criteria version 4, with a possible range of 0–4)	MD 0.00 95% CI -0.43 to 0.43, p = 1.00

*Note*: *CI*, Confidence Interval; *CTCAE*, Common Terminology Criteria for Adverse Events; *HR*, Hazard Ratio; *LED*, Light Emitting Diode photo-modulation; *MD*, Mean Difference; *OR*, Odds Ratio; *RISR*, Radiation Induced Skin Reactions; *RTOG*, Radiation Therapy Oncology Group; *SD*, Standard Deviation; *SE*, Standard Error.

### Prevention of radiation-induced skin reactions

#### Oral systemic therapies

**Figure 5:**
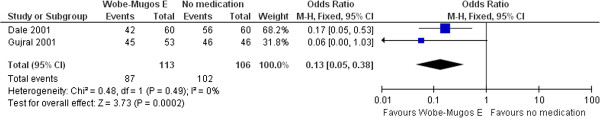
Forest plot of comparison: Whole-Mugos E vs usual care (no medication), Outcome: Development of RISR (Yes/No).

One fixed effect meta-analysis including 219 participants from two unblinded RCTs [32,33] suggested that the odds of developing a RISR was 87% lower for people receiving oral Wobe-Mugos E (proteolytic enzymes containing 100 mg papain, 40 mg trypsin, and 40 mg chymotrypsin) (three tablets) than no medication (OR 0.13, 95% CI 0.05 to 0.38, p < 0.0005) (see Figure 5). The two trials [32,33] had slight variation of daily dosage and days of administration before commencement of radiation treatment. However, one small unblinded trial of 74 participants [30] reported that oral pentoxifylline was ineffective for preventing the development of RISR.

#### Washing practices and deodorant/antiperspirant use

**Figure 6:**
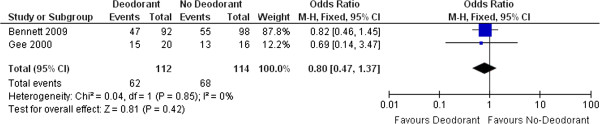
Forest plot of comparison: Deodorant vs non-deodorant, Outcome: Development of a RISR.

One trial [37], using a non-blinded design with 99 participants, provided results concerning how washing practices might contribute to the prevention of a RISR. This study did not find any benefits of washing with soap for preventing a RISR. A fixed effect meta-analysis involving 226 participants from two unblinded trials also did not find any difference between using deodorant or not in the development of a RISR [38,39] (see Figure 6).

#### Steroidal topical treatment

Four trials [42-45] evaluated the effectiveness of a range of topical steroidal treatments for preventing the development RISR. Two trials [44,45] reported negative results, suggesting betamethasone cream and 0.1% mometasone furoate cream, in comparison with no treatment or placebo, were not effective for preventing the development RISR. Another trial [42] also did not find any difference in this outcome when comparing 1% hydrocortisone cream and 0.05% clobetasone burtrate. A small trial of 20 participants [43] reported statistically significant results favouring topical preparation containing prednisolone with neomycin compared to no treatment (OR 0.07, 95% CI 0.01 to 0.84, p = 0.04). However, the results need to be considered with caution as the latter trials [42,43] had small sample sizes with wide confidence intervals.

#### Non-steroidal topical treatment

Hyaluronic acid and trolamine were separately reported to be ineffective for preventing the development of RISR in two trials [48,55]. Another trial [50] also did not find any difference between two non-steroidal “restitutio restructuring” preparations in this outcome. A non-blinded trial of 126 participants [57] reported the effectiveness of Lian Bai liquid for preventing the development of RISR. Lian Bai liquid is consisted of Huang Lian (Rhizoma Coptidis) 15 g and Huang Bai (Cortex Phellodendri) 15 g soaked in 800 ml of water. According to the results, the odds of developing a RISR were lower for participants receiving Lian Bai liquid than those who received no treatment (OR 0.04, 95% CI 0.01 to 0.12, p < 0.00001).

#### LED treatment versus Sham treatment

One small blinded RCT including 33 participants [74] reported no benefits of LED for reducing the development of RISR.

### Treatment of radiation-induced skin reactions

#### Oral systemic therapies

**Figure 7:**
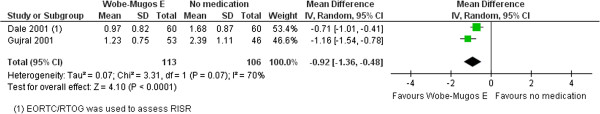
Forest plot of comparison: Wobe-Mugos E vs usual care (no medication), Outcome: Maximum levels of skin toxicity as measure by EORTC/RTOG, with a possible range of 0-4.

With regards to oral systemic therapies, one random effects meta-analysis including 219 participants from two unblinded RCTs [32,33] suggested that the maximum extent of RISR severity was significantly lower in those who received oral Wobe-Mugos E (MD -0.92, 95% CI -1.36 to -0.48, p < 0.0001) (see Figure 7). Both trials used different dosing per day for the intervention arm. In one trial [32], participants were prescribed three tablets of oral Wobe-Mugos E four times a day, whereas participants in the other trial [33] were prescribed three tablets of oral Wobe-Mugos E three times a day. The findings from a blinded RCT including 97 participants [35] reported that 25 mg oral zinc supplementation (three capsules daily) was effective for reducing the RISR severity at the end of radiation treatment (MD -0.50, 95% CI -0.58 to -0.42, p < 0.00001). Of all trials evaluating the effectiveness oral systemic therapies, none of the trials compared the effectiveness between dosing and administration period (e.g. start of radiation treatment to a certain number of additional days post radiation treatment). The effectiveness of oral pentoxifylline, oral antioxidant, or oral sucralfate suspensions for reducing symptoms related to radiation treatment cannot be determined with the available data.

#### Washing practices and deodorant/antiperspirant use

In terms of washing practices, washing with soap was more effective than not washing at all for improving a number of outcomes including lower incidence of desquamation [37], lower levels of erythema at week six [36] (MD-0.40 95% CI -0.77 to -0.03, p = 0.03) and lower levels of desquamation at the end of treatment (week six) (MD -0.47, 95% CI -0.83 to -0.11, p = 0.01) and two weeks follow-up (week eight) (MD -0.82, 95% CI -1.16 to -0.48, p < 0.00001) [36]. Besides, the same three-armed trial [36] reported that washing with soap was also more effective than washing with water for reducing the levels of desquamation at the end of treatment (week six) (MD -0.59, 95% CI -0.94 to -0.24, p = 0.001) and the two-week follow-up (week eight) (MD -0.62, 95% CI -0.96 to -0.28, p = 0.0004). Levels of itch and erythema at week eight of radiation treatment were also lower in the soap group. When comparing washing with water or with soap, the only statistically significant difference observed was erythema at week eight (MD -0.44, 95% CI -0.72 to -0.16, p = 0.001) [36].

While it was thought that deodorant (both metallic and non-metallic) might have an undesirable effect on the skin, there were no differences in a number of RISR outcomes between those who wore deodorant or not as reported by four trials [38-41]. Three studies [38-40] compared non-metallic deodorant with no deodorant, while one study [41] compared a deodorant containing 21% of aluminium with no deodorant. A trial of 84 participants [40] reported that deodorant could significantly reduce the incidence of sweating at the end of the radiation treatment, but not at the two-week follow-up (OR = 0.70, 95% CI -0.25 to 1.99, p = 0.51).

#### Steroidal topical treatment

A blinded RCT of 166 participants reported that topical 0.1% mometasone furoate cream was not superior to placebo for reducing the maximum level of RISR or RISR level at two weeks after the end of treatment [44]. One small RCT reported that 0.1% betamethasone cream was effective for reducing the maximum level of RISR when compared to placebo (MD -1.62, 95% CI -2.03 to -1.21, p < 0.00001), and for reducing RISR severity at seven weeks (two week follow-up) of radiation treatment (MD -0.55, 95% CI -0.71 to -0.39, p < 0.00001) [45]. One RCT [46], with a small sample size of 21 participants, reported that 0.1% methylprednisolone was more effective than dexpanthenol for reducing the levels of RISR at the two-week follow-up post radiation treatment (p < 0.00001).

#### Non-steroidal topical treatment

Two small-to-medium size blinded placebo RCTs [55,56] reported the effectiveness of hyaluronic acid compared with placebo for reducing the level of RISR severity at the end of radiation treatment [56] (MD -0.73, 95% CI -1.04 to -0.42, p < 0.00001), at four weeks after radiation treatment completion [56] (MD -0.35, 95% CI -0.68 to -0.02, p = 0.04) and the maximum level of RISR [55] (MD-0.95, 95% CI -1.23 to -0.67, p < 0.00001). Additionally, there were no statistically significant adverse effects of hyaluronic acid compared to placebo [55,61]. However, one trial [54] did not find that hyaluronic acid was effective for reducing the incidence of severe pain or quality of life at week four of radiation treatment. One three-arm RCT of a total of 68 participants [63] compared two formulations with each other, and with no treatment. This trial reported that formulation A topical cream (capprais spinosa, opuntia coccinellifera and olive leaf extract) was more effective than formulation B (non-steroidal water based emulsion) for reducing the maximum RISR level and the erythema index over a period of eight weeks. Moreover, either formation was more effective than no treatment for reducing these two outcomes [63].

**Figure 8:**
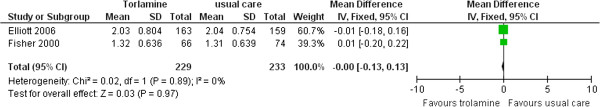
**Forest plot of comparison: trolamine vs control.** Outcome: Maximum levels of skin toxicity as measure by EORTC/RTOG, with a possible range of 0-4.

According to the results of a fixed-effect meta-analysis, trolamine was reported to not be more effective than usual care (institutional preference) for reducing the maximum level of RISR (MD 0.00, 95% CI -0.13 to 0.13, p = 0.97) [48,49] (see Figure 8). When it was compared with placebo, one trial [51] (n = 102) reported that participants found it easier to use (MD 1.12, 95% CI 0.56 to 1.68, p < 0.00001) and were more satisfied with trolamine (MD 0.44, 95% CI 0.01 to 0.87, p = 0.04). However, another non-blinded RCT of 254 participants [60] reported that significantly more patients in this trial rated calendula more difficult to apply than trolamine (OR 7.68, 95% CI 3.07 to 19.17, p < 0.0001). One multi-treatment arm trial [51] also reported no statistically significant benefits of RadiaCare Gel and Aquaphor ointment when individually compared to placebo for reducing RISR, although the participants were more satisfied with these two interventions, compared with placebo topical preparations. According to the findings from the analyses in this review, we were unable to find any benefits of aloe vera gel and dexpanthenol, when compared with placebo or no treatment. While a number of trials individually reported statistically significant benefits of using qingdiyou medication, wheatgrass extract cream, and sucralfate cream for reducing RISR, we were unable to conduct analysis due to inadequate data despite attempts of contacting the trial authors.

#### Dressings

Two small non-blinded RCTs [69,70], with less than 40 participants in each trial, examined the effectiveness of gentian violet dressings on time to healing when comparing with a hydrogel dressing and non-adherent dressing. The smaller trial (n = 20) reported results favouring the hydrogel dressing (HR 7.95, 95% CI 2.20-28.68, p = 0.002), with other trial (n = 39) reporting non-significant difference between the gentian violet dressing group and non-adherent dressing group (HR 0.73, 95% CI 0.52-1.03, p = 0.07). One trial [69] also reported there was no statistically significant difference between the two dressings in adverse effects. A small non-blinded RCT of 40 participants [71] reported that silver nylon dressing was more effective than standard care in reducing the level of RISR severity at the end of radiation treatment (MD = -0.86, 95% CI -1.59 to -0.13, p = 0.02).

#### LED treatment versus Sham treatment

One small blinded RCT including 33 participants [74] reported no benefits of LED for reducing the levels of RISR at the end of radiation treatment (week five).

## Discussion

The methodological quality of most of the RCTs was fair, at best, with limitations in a number of domains. Another limitation was the small number of studies per intervention to perform meta-analyses. The largest meta-analysis in this review only had 229 participants in the active treatment arm and 223 participants in the control arm. In some cases, meta-analysis was considered inappropriate as the constituents of some topical applications varied between studies, as did the placebo creams; in trials that compared dressings with standard care, the dressings were made of different materials. Consequently, the evidence was restricted to indirect comparisons between these varied interventions. Additionally, as some therapies were only tested on particular anatomical sites in populations with mixed gender, age groups, and radiation techniques and dosage, the evidence may be regarded as indirect for other areas exposed to radiation. Taken together, these limitations restrict confident decision making with regard to the use of any of the included products to prevent RISR. We were able to combine results for only four comparisons. Heterogeneity was absent in three of these but high (I^2^ = 70%) in the comparison between Wobe-Mogus E versus usual care. The decision to combine results for trials of Wobe-Mogus E, despite the high level of heterogeneity, was based on the fact that results of both trials favoured the intervention product and that heterogeneity was most likely due to differences in dosing regimens. Confidence intervals were wide for most of the comparisons included in this review. The wide CIs are probably explained by small sample sizes, indicating a high level of uncertainty around the effect sizes. Further research is, therefore, very likely to have an important impact on the confidence of the estimate of effect for most of the included interventions.

Over the duration of the review, the research team made a decision based on clinical reasons to include additional outcomes concerning the time points of the RISR associated outcomes. The original time points were too rigid and not necessarily the most clinically relevant, it was clinically important for clinicians to assess RISR using the maximum RISR levels and the levels of RISR at the end of treatment and the last follow-up. The maximum level of RISR represents the worst reaction associated with a given intervention or no intervention at all. RISR levels at the end of the radiation treatment and the last follow-up were expected to be clinically relevant, and allowed the inclusion of studies with differing end of treatment and follow up times. These ‘follow-up’ time points are often the last occasions of care for radiation oncologists, radiation oncology nurses, and radiation therapists.

Despite the potential limitations stated above, our review represents the highest quality and most up-to-date systematic review in this area and includes 47 RCTs of 5,688 participants. Whilst there have been a number of systematic reviews published about the topic of RISRs, these reviews have limited capacity for translation into clinical practice due to significant methodological and design shortfalls [24]. For example, many of these systematic reviews are out-dated and limit studies for inclusion to those written in the English language. In light of these common shortfalls across pre-existing publications on this topic, this paper aims to provide an up-to-date systematic review and meta-analysis of interventions that are currently utilised within clinical practice to prevent and manage RISRs.

The results of review highlight that none of the studies measured all outcomes of interest. Some non-clinical outcomes (such as cost or costs associated treatment delay and labour) can be important for clinical decision making. Future trials should include these important outcomes.

Since the search of this review was conducted (11/2012), at least three trials [75-77] relevant to the research question of interest have been published. An RCT of 411 patients by Sharp et al. reported no significant difference between Calendula Weleda® and Essex cream® in reducing RISR [76]. Another double-blind RCT of 318 patients conducted by Graham et al. also reported negative findings in their RCT on the effects of a moisturizing durable barrier cream and glycerine cream [75]. With regards to dressings, a small unblinded RCT (n = 88) reported a significant reduction in time to heal when Mepilex Lite dressing was used in comparison with the usual care group [77]. The results of this review suggest that the evidence base is extremely wide, with many interventions tested. However, evidence for any particular product remains thin, with very few trials examining the effects of any single intervention. Therefore, we do not expect the direction of recommendation to change with the additional trials published over this period. However, it is important that any subsequent trials should be included in the future updates of the systematic review. Multiple high quality trials comparing promising interventions are urgently needed.

## Conclusion

This review found no strong evidence of effect for any of the included trial products in reducing RISR. In practical terms, the use of soap for washing did not show higher levels of RISR compared with washing with water alone, nor did the use of deodorants affect RISR levels. Consequently, clinicians may advise patients to gently wash affected areas with soap and water during their treatment and that non-metallic deodorant use is not contraindicated. Further research is required to establish whether metallic deodorants should be used. High quality, well-powered studies are required to provide additional evidence for the effectiveness of various products. In particular, efforts should focus on products which show promise, such as Wobe-Mugos E and oral zinc for reducing the RISR severity. Further research is also required to understand which groups of patients may benefit from topical corticosteroid treatment. There is also a lack of well-powered RCTs comparing different types of dressings for those who develop a desquamation requiring dressings. Importantly, future trials should include outcomes such as skin-specific quality of life, and costs.

## Abbreviations

CI: Confidence interval; MD: Mean difference; NNT: Numbers needed to treat; OR: Odds ratio; RISR: Radiation-induced skin reaction; RTOG: Radiation Therapy Oncology Group; RCT: Randomized-controlled trial; UV: Ultraviolet

## Competing interests

The authors declare that they have no competing interests.

## Authors’ contributions

RC co-ordinated contributions from co-authors. All authors took part in drafting the protocol. Search strategies were developed by RC and JW. Searches were conducted by Elizabeth Doney, the Trial Search Coordinator of the Cochrane Skin Group. RC and JW identified relevant titles and abstracts from searches. Full-text copies of trial reports were obtained by RC. Included trials were selected by RC and JW. Data was extracted from trials by RC, JW, AM, KN and PH. Data entry into RevMan as well as was carried out by RC. Analysis and interpretation of analysis was carried out by RC, JW and LM. All authors were involved in drafting and approving the final review, with RC being the co-ordinating author. All authors read and approved the final manuscript.

## Pre-publication history

The pre-publication history for this paper can be accessed here:

http://www.biomedcentral.com/1471-2407/14/53/prepub

## Supplementary Material

Additional file 1 Data sources and searches. Click here for file

Additional file 2 Characteristics of excluded studies. Click here for file

Additional file 3 Data extraction sheet. Click here for file

Additional file 4 Characteristics of included studies. Click here for file
