# Supplementary material for: Prevention and treatment of acute radiation-induced skin reactions: a systematic review and meta-analysis of randomized controlled trials
Source: BMC Cancer. 2014 Jan 31;14:53. doi: 10.1186/1471-2407-14-53 (PMC3909507; doi:10.1186/1471-2407-14-53)
Supplement: Additional file 1 — Data sources and searches. [file 1471-2407-14-53-S1.doc]

**Additional File -1 Data Sources and Searches**

**Database Searches**

*1 CENTRAL search strategy*

#1 MeSH descriptor Radiation Injuries explode all trees
#2 MeSH descriptor Fibrosis explode all trees
#3 MeSH descriptor Erythema explode all trees
#4 MeSH descriptor Radiodermatitis explode all trees
#5 radiodermatitis
#6 (radiation next induced next skin next reaction)
#7 erythema
#8 desquamation
#9 ulceration
#10 redness
#11 fibrosis
#12 burning
#13 rash
#14 itch
#15 swell
#16 MeSH descriptor Radiotherapy explode all trees
#17 MeSH descriptor Radiation Oncology explode all trees
#18 (radiother* or radiat* or irradiat* or radiochemo* or chemoradiat*)
#19 "skin reaction" or "skin alteration" or "skin toxic" or "skin change"
#20 (#1 OR #2 OR #3 OR #4 OR #5 OR #6 OR #7 OR #8 OR #9 OR #10 OR #11 OR #12 OR #13 OR #14 OR #15 OR #19)
#21 (#16 OR #17 OR #18)
#22 lymphoma* or sarcoma* or ewing* or osteosarcom* or wilms or nephroblastom* or neuroblastom* or rhabdomyosarcom* or teratom* or hepatom* or hepatoblastom* or pnet or medulloblastom* or retinoblastom* or meningiom* or gliom*
#23 "neuroectodermal tumor* primitive" or "t cell" or "b cell" or "brain tumor*" or "brain tumour*" or "brain neoplasm*" or "central nervous system neoplam*" or "central nervous system tumour*" or "central nervous system tumor*" or "brain cancer" or "brain neoplasm" or "intracranial neoplasm" or "leukemia lymphocytic acute"
#24 MeSH descriptor Neoplasms explode all trees
#25 cancer or oncolog* or neoplasm* or carcinom* or tumor* or tumour* or malignan* or hematooncological or hematolo* or "hemato oncological"
#26 (#22 OR #23 OR #24 OR #25)
#27 (#20 AND #21 AND #26)

*2 MEDLINE search strategy (OVID)*

1. exp Radiodermatitis/ or radiodermatitis.mp.
2. radiation induced skin reaction.mp.
3. erythema.mp. or exp Erythema/
4. Desquamation.mp.
5. ulceration.mp.
6. redness.mp. or exp Skin Pigmentation/
7. exp Fibrosis/ or fibrosis.mp.
8. burning.mp.
9. rash.mp.
10. swell$3.mp.
11. itch$.mp.
12. (skin reaction$ or skin alter$ or skin toxic$ or skin change$).mp.
13. exp Radiation Injuries/
14. 1 or 2 or 3 or 4 or 5 or 6 or 7 or 8 or 9 or 10 or 11 or 12 or 13
15. exp Radiotherapy/
16. exp Radiation Oncology/
17. (radiother$ or radiat$ or irradiat$ or radiochemo$ or chemoradiat$).mp.
18. 15 or 16 or 17
19. (cancer$ or oncolog$ or neoplasm$ or carcinom$ or tumor$ or tumour$ or malignan$ or hematooncological or hematolo$).mp.
20. hemato oncological.mp.
21. exp Neoplasms/
22. (lymphom$ or sarcom$ or ewing$ or osteosarcom$ or wilms or nephroblastom$ or neuroblastom$ or rhabdomyosarcom$ or teratom$ or hepatom$ or hepatoblastom$ or PNET or medulloblastom$ or retinoblastom$ or meningiom$ or gliom$).mp.
23. (neuroectodermal tumor$ primitive or T-cell or B-cell or brain tumor$ or brain tumour$ or brain neoplasm$ or central nervous system neoplasm$ or central nervous system tumor$ or central nervous system tumour$ or brain cancer$ or brain neoplasm$ or intracranial neoplasm$ or leukemia lymphocytic acute).mp.
24. 19 or 20 or 21 or 22 or 23
25. randomized controlled trial.pt.
26. controlled clinical trial.pt.
27. randomized controlled trial.pt.
28. controlled clinical trial.pt.
29. randomized.ab.
30. placebo.ab.
31. clinical trials as topic.sh.
32. randomly.ab.
33. trial.ti.
34. 27 or 28 or 29 or 30 or 31 or 32 or 33
35. exp animals/ not humans.sh.
36. 34 not 35
37. 14 and 18 and 24 and 36

*3 EMBASE search strategy (OVID)*

1. radiodermatitis.mp. or exp radiation dermatitis/
2. radiation induced skin reaction.mp.
3. erythema.mp. or exp ERYTHEMA/
4. DESQUAMATION/ or desquamation.mp.
5. ulceration.mp.
6. redness.mp. or exp SKIN REDNESS/
7. exp FIBROSIS/ or fibrosis.mp.
8. burning.mp.
9. exp RASH/ or rash.mp.
10. swell$3.mp.
11. itch$.mp.
12. (skin adj (reaction$ or alter$ or toxic$ or change$)).mp.
13. exp radiation injury/
14. 1 or 2 or 3 or 4 or 5 or 6 or 7 or 8 or 9 or 10 or 11 or 12 or 13
15. exp RADIOTHERAPY/
16. radiation oncology.mp.
17. (radiother$ or radiat$ or irradiat$ or radiochemo$ or chemoradiat$).mp.
18. 15 or 16 or 17
19. (cancer$ or oncolog$ or neoplasm$ or carcinom$ or tumor$ or tumour$ or malignan$ or hematooncological or hematolo$).mp.
20. hemato oncological.mp.
21. exp neoplasm/
22. (lymphom$ or sarcom$ or ewing$ or osteosarcom$ or wilms or nephroblastom$ or neuroblastom$ or rhabdomyosarcom$ or teratom$ or hepatom$ or hepatoblastom$ or PNET or medulloblastom$ or retinoblastom$ or meningiom$ or gliom$).mp.
23. (neuroectodermal tumor$ primitive or T-cell or B-cell or brain tumor$ or brain tumour$ or brain neoplasm$ or central nervous system neoplasm$ or central nervous system tumor$ or central nervous system tumour$ or brain cancer$ or brain neoplasm$ or intracranial neoplasm$ or leukemia lymphocytic acute).mp.
24. 19 or 20 or 21 or 22 or 23
25. crossover procedure.sh.
26. double-blind procedure.sh.
27. single-blind procedure.sh.
28. (crossover$ or cross over$).tw.
29. placebo$.tw.
30. (doubl$ adj blind$).tw.
31. allocat$.tw.
32. trial.ti.
33. randomized controlled trial.sh.
34. random$.tw.
35. or/25-34
36. (ANIMAL/ or NONHUMAN/ or ANIMAL EXPERIMENT/) and HUMAN/
37. ANIMAL/ or NONHUMAN/ or ANIMAL EXPERIMENT/
38. 37 not 36
39. 35 not 38
40. 14 and 18 and 24 and 39
41. remove duplicates from 40

*4 PsycINFO search strategy (OVID)*

1. double-blind.tw.
2. random$ assigned.tw.
3. control.tw.
4. 1 or 2 or 3
5. exp Radiation Therapy/ or radiation.mp.
6. cancer.mp. or exp Neoplasms/
7. skin.mp.
8. 5 and 6 and 7
9. 4 and 8

NB Lines 1-3 of this strategy are a therapy filter for PsycINFO (OVID) created by the [Health Information Research Unit](http://hiru.mcmaster.ca/hiru/HIRU_Hedges_PsycINFO_Strategies.aspx) at McMaster University.

*5 CINAHL search strategy (EBSCO)*

S1      (MH "Radiodermatitis") OR radiodermatitis
S2      erythema or desquamation or ulceration or redness or fibrosis or burning or rash or swell or itch
S3      radiation induced skin reaction
S4      "skin reaction*" or "skin alter*" or "skin toxic*" or "skin change*"
S5      (MH "Erythema+")
S6      (MH "Fibrosis")
S7      ((MH "Fibrosis")) and (S1 and S2 and S3 and S4 and S5 and S6)
S8      ((MH "Fibrosis")) and (S1 and S2 and S3 and S4 and S5 and S6)
S9      S1 or S2 or S3 or S4 or S5 or S6 or S7 or S8
S10     (MH "Radiotherapy+")
S11     (MH "Radiation Oncology")
S12     radiother* or radiat* or irradiat* or radiochemo* or chemoradiat*
S13     s10 or s11 or s12
S14     (MH "Neoplasms+")
S15     cancer* or oncolog* or neoplasm* or carcinom* or tumor* or tumour* or malignan* or hematooncological or hematolo* or lymphoma* or sarcoma* or ewing* or osteosarcoma* or wilms or nephroblastoma* or neuroblastoma* or rhabdomysarcoma*or teratom* or hepatom* or hepatoblastom* or pnet or medulloblastom* or retinoblastom* or meningiom* or gliom* or "hemato oncological"
S16     "neuroectodermal tumor* primitive" or "t cell" or "b cell" or "brain tumor" or "brain tumour" or "brain neoplasm" or "central nervous system neoplasm*" or "central nervous system tumour" or "central nervous system tumor" or "brain cancer" or "brain neoplasm" or "intracranial neoplasm*" or "leukemia lymphocytic acute"
S17     S14 or S15 or S16
S18     S9 and S13 and S17
S19     (MH "Clinical Trials+")
S20     PT clinical trial
S21     TX (clinic* n1 trial*)
S22     (MH "Random Assignment")
S23     TX random* allocat*
S24     TX placebo*
S25     (MH "Placebos")
S26     (MH "Quantitative Studies")
S27     TX allocat* random*
S28     "randomi#ed control* trial*"
S29     Singl* n5 blind* or doubl* n5 blind* or trebl* n5 blind* or tripl* n5 mask* or singl* n5 mask* or doubl* n5 mask* or trebl* n5 mask* or tripl* n5 mask*
S30     S19 or S20 or S21 or S22 or S23 or S24 or S25 or S26 or S27 or S28 or S29
S31     S18 and S30

*6 LILACS search strategy*

((Pt RANDOMIZED CONTROLLED TRIAL OR Pt CONTROLLED CLINICAL TRIAL OR Mh RANDOMIZED CONTROLLED TRIALS OR Mh RANDOM ALLOCATION OR Mh DOUBLE-BLIND METHOD OR Mh SINGLE-BLIND METHOD OR Pt MULTIcentre STUDY) OR ((tw ensaio or tw ensayo or tw trial) and (tw azar or tw acaso or tw placebo or tw control$ or tw aleat$ or tw random$ or (tw duplo and tw cego) or (tw doble and tw ciego) or (tw double and tw blind)) and tw clinic$)) AND NOT ((CT ANIMALS OR MH ANIMALS OR CT RABBITS OR CT MICE OR MH RATS OR MH PRIMATES OR MH DOGS OR MH RABBITS OR MH SWINE) AND NOT (CT HUMAN AND CT ANIMALS)) [Words] and radiation or radiacion [Words] and skin or piel [Words]

**Trial Registers**

We searched the following trial registers listed below.

- The metaRegister of Controlled Trials ([http://www.controlled-trials.com](http://www.controlled-trials.com/))
- The U.S. National Institutes of Health ongoing trials register ([http://www.clinicaltrials.gov](http://www.clinicaltrials.gov/)).
- The Australian and New Zealand Clinical Trials Registry ([http://www.anzctr.org.au](http://www.anzctr.org.au/)).
- The World Health Organization International Clinical Trials Registry platform (<http://www.who.int/trialsearch>).
- The Ongoing Skin Trials register (<http://www.nottingham.ac.uk/ongoingskintrials>).

**Reference Lists**

We searched the reference lists reported in relevant reviews and studies which were not identified via electronic searches.

We also scanned the contents pages of the following journals (from inception to November 2012) for articles about interventions that aim to prevent or manage RISRs, and any abstracts from relevant conference proceedings. These included:

- Radiation Oncology;
- International Journal of Radiation Oncology;
- Biology and Physics, Radiotherapy and Oncology;
- Journal of Medical Imaging and Radiation Oncology;
- Journal of Pain and Symptom Management;
- Oncology Nursing Forum;
- Cancer Nursing;
- Supportive Care in Cancer;
- Journal of Alternative Therapies;
- Journal of Integrative Cancer Therapies;
- The Multinational Association of Supportive Care in Cancer International Symposium;
- The Clinical Oncological Society of Australia Annual Scientific Meetings;
- The Oncology Nursing Society Annual Congresses; and
- The Cancer Nurses Society of Australia Winter Congresses.

**Correspondence**

We contacted first authors of the five most recently published included studies for advice about other potentially relevant studies. We also searched the ProQuest Dissertations and Theses database for grey literature.
