# Supplementary material for: Prevention and treatment of acute radiation-induced skin reactions: a systematic review and meta-analysis of randomized controlled trials
Source: BMC Cancer. 2014 Jan 31;14:53. doi: 10.1186/1471-2407-14-53 (PMC3909507; doi:10.1186/1471-2407-14-53)
Supplement: Additional file 2 — Characteristics of excluded studies. [file 1471-2407-14-53-S2.doc]

**Additional File 2- Table of characteristics of excluded studies in alphabetical order by author:**

| **Study Reference** | **Reason for Exclusion** |
| --- | --- |
| **Abbas, 2012** | This was a RCT evaluating an intervention for managing RISR., but this study did not report on the outcome of interest of this review. |
| **Angelakis, 1973** | This is a non RCT. |
| **Anne, 2002** | This study does not fit the objective of evaluating interventions for managing RISR. |
| **Antonadou, 2002** | This study does not fit the objective of evaluating interventions for managing RISR. This study examines the effects on mucositis. |
| **Bain, 2005** | This is a quasi-experimental study. |
| **Balzarini, 2000** | This was a RCT evaluating an intervention for managing RISR., but this study did not report on the outcome of interest of this review. |
| **Bostrom, 2001** | This was a RCT evaluating an intervention for managing RISR., but this study did not report on the outcome of interest of this review. |
| **Bourgeois, 2008** | This study does not fit the objective of evaluating interventions for managing RISR. This study examined the effect of massage on cutaneous fibrosis from 6 to 16 months. |
| **Ceconi, 2009** | This is a non RCT. |
| **Costa, 1974** | This is a non RCT. |
| **Currie, 2006** | This is a non RCT. |
| **Das, 2011** | This study does not fit the objective of evaluating interventions for managing RISR. |
| **Diggelmann, 2010** | This is a non RCT. |
| **Dunst, 2000** | This is a non RCT. |
| **Evensen, 2001** | This was a RCT evaluating an intervention for managing RISR., but this study did not report on the outcome of interest of this review. |
| **Fenig, 2001** | This is a quasi-experimental study. |
| **Freedman, 2006** | This study concerns radiation treatment technique, rather than RISR management. And this is a non RCT. |
| **Gupta, 2009** | This study concerns radiation treatment technique, rather than RISR management. |
| **Halperin, 1993** | This was a RCT evaluating an intervention for managing RISR., but this study did not report on the outcome of interest of this review. |
| **Harsolia, 2007** | This is a non RCT. |
| **Herst, 2010** | This is a non RCT. |
| **Hu, 2005** | This was a RCT evaluating an intervention for managing RISR., but this study did not report on the outcome of interest of this review. |
| **Korabek, 1994** | Insufficient information to assess its eligibility for this review. |
| **Kouvaris, 2001** | This is a non RCT. |
| **Kouvaris, 2002** | This is a non RCT. |
| **Livi, 2010** | This study concerns radiation treatment technique, rather than RISR management. |
| **Lokkevik, 1996** | This was a RCT evaluating an intervention for managing RISR., but this study did not report on the outcome of interest of this review. |
| **Macmillan, 2007** | This was a RCT evaluating an intervention for managing RISR., but this study did not report on the outcome of interest of this review. |
| **Maiche, 1991** | This was a RCT evaluating an intervention for managing RISR., but this study did not report on the outcome of interest of this review. |
| **Mak, 2000** | This was a RCT evaluating an intervention for managing RISR., but this study did not report on the outcome of interest of this review. |
| **Matceyevsky, 2007** | This is a non RCT. |
| **Merchant, 2006** | This is a non RCT. |
| **Miko, 2005** | This was a RCT evaluating an intervention for managing RISR., but this study did not report on the outcome of interest of this review. |
| **Mirri, 2009** | This is a non RCT. |
| **Momm, 2003** | This is a non RCT. |
| **Moolenaar, 2006** | This is a non RCT. |
| **Neben-Wittich, 2011** | This is a non-RCT. This study investigated the correlation between skin-related quality of life and dermatitis outcomes. |
| **Nystrom, 2007** | This is a non RCT. |
| **Olivotto, 1996** | This study does not fit the objective of evaluating interventions for managing RISR. |
| **Omidvari, 2011a** | This was a RCT evaluating an intervention for managing RISR., but this study did not report on the outcome of interest of this review. |
| **Patwardhan, 2002** | This study did not measure the outcomes of interest in this review. |
| **Pignol, 2008** | This study concerns radiation treatment technique, rather than RISR management. |
| **Pignol, 2010** | This is a non RCT. |
| **Pinnix, 2010** | This was a RCT evaluating an intervention for managing RISR., but this study did not report on the outcome of interest of this review. |
| **Pischnamazzedah, 1983** | This is a non RCT. |
| **Porock, 1999** | This is a non RCT. |
| **Robinson, 1987** | Insufficient information to determine the eligibility for inclusion in this review. |
| **Rudd, 2002** | This was a RCT evaluating an intervention for managing RISR., but this study did not report on the outcome of interest of this review. |
| **Ryan, 2010** | This is a non RCT. |
| **Roper, 2004** | This is a quasi-RCT. |
| **Schreck, 2002** | This is a non RCT. |
| **Shoma, 2010** | This was a RCT evaluating an intervention for managing RISR., but this study did not report on the outcome of interest of this review. |
| **Shukla, 2006** | This was a RCT evaluating an intervention for managing RISR., but this study did not report on the outcome of interest of this review. |
| **Szumacher, 2001** | This is a non RCT. |
| **Vuong, 2004** | This is a non RCT. |
| **Westbury, 2000** | This was a RCT evaluating an intervention for managing RISR., but this study did not report on the outcome of interest of this review. |
| **Wollina, 2002** | This is a non RCT. |
| **Zhou, 1993** | This is a non RCT. |

1. Abbas H, Bensadoun R J: **Trolamine emulsion for the prevention of radiation dermatitis in patients with squamous cell carcinoma of the head and neck**. *Supportive Care in Cancer* 2012, **20**:185-190.

2. Angelakis P, Papavasiliou C, Elias C: **Twice per week treatment versus five times per week. A radiotherapeutic clinical trial**. *The British Journal of Radiology* 1973, **46**(545):350-353.

3. Anné P R: **Phase II trial of subcutaneous amifostine in patients undergoing radiation therapy for head and neck cancer**. *Seminars in Oncology* 2002, **29**(6 Suppl 19):80-83.

4. Antonadou D: **Radiotherapy or chemotherapy followed by radiotherapy with or without amifostine in locally advanced lung cancer**. *Seminars in Radiation Oncology* 2002, **12**(1 Suppl 1):50-58.

5. Bain G, Kuwahata H, Raymond B, Foster R: **Tea Tree/ Hydrogel Dressing Used in Wound Care: a repeated measures comparative study of a tea-tree oil and a pawpaw cream dressing**. In: *Australian Government Report.* Rural industries Research and Development Corporation; 2005: 26-26.

6. Balzarini A, Felisi E, Martini A, De Conno F: **Efficacy of homeopathic treatment of skin reactions during radiotherapy for breast cancer: a randomised, double-blind clinical trial**. *The British Homoeopathic Journal* 2000, **89**(1):8-12.

7. Boström A, Lindman H, Swartling C, Berne B, Bergh J: **Potent corticosteroid cream (mometasone furoate) significantly reduces acute radiation dermatitis: results from a double-blind, randomised study**. *Radiotherapy and Oncology* 2001, **59**(3):257-265.

8. Bourgeois J F, Gourgou S, Kramar A, Lagarde J M, Guillot B: **A randomised, prospective study using the LPG technique in treating radiation-induced skin fibrosis: clinical and profilometric analysis**. *Skin research and technology* 2008, **14**(1):71-76.

9. Ceconi, Guido A, Cammelli S, Bunkheila F, Micucci M, Pepe A, et al: **Cutaneous reaction prevention with the use of "Neoviderm Skin Emulsion" in head and neck external beam radiotherapy (EBRT) and preioperative-Brachytherapy (BRT) for breast cancer**. *TUMORI* 2009, **Supplementto 8**(n1):s10-s10.

10. Costa Martins J E, Pozetti G L, Sodre M: **Effects of psoralen and bergapten on irradiated skin**. *International Journal of Dermatology* 1974, **13**(3):124-128.

11. Currie G, Wheat J: **Wheatgrass extract as a topical skin agent for acute radiation skin toxicity in breast radiation therapy**. *Journal of the Australian Traditional-Medicine Society* 2006, **12**(1):7-11.

12. Das D, Agarwal S, Chandola HM: **Protective effect of Yashtimadhu (Glycyrrhiza glabra) against side effects of radiation/chemotherapy in head and neck malignancies**. *AYU* 2011, **32**(2):196-199.

13. Diggelmann K V, Zytkovicz A E, Tuaine J M, Bennett N C, Kelly L E, Herst P M: **Mepilex Lite dressings for the management of radiation-induced erythema: a systematic inpatient controlled clinical trial**. *British Journal of Radiology* 2010, **83**:971-978.

14. Dunst J, Semlin S, Pigorsch S, Muller A C, Reese T: **Intermittent use of amifostine during postoperative radiochemotherapy and acute toxicity in rectal cancer patients**. *Strahlentherapie und Onkologie* 2000, **176**(9):416-421.

15. Evensen J F, Bjordal K, Jacobsen A B, Løkkevik E, Tausjø J E: **Effects of Na-sucrose octasulfate on skin and mucosa reactions during radiotherapy of head and neck cancers--a randomised prospective study**. *Acta Oncologica (Stockholm, Sweden)* 2001, **40**(6):751-755.

16. Fenig E, Brenner B, Katz A, Sulkes J, Lapidot M, Schachter J, et al: **Topical Biafine and Lipiderm for the prevention of radiation dermatitis: a randomised prospective trial**. *Oncology Report* 2001, **8**(2):305-309.

17. Freedman G M, Anderson P R, Li J, Eisenberg D F, Hanlon A L, Wang L, et al: **Intensity modulated radiation therapy (IMRT) decreases acute skin toxicity for women receiving radiation for breast cancer**. *American Journal of Clinical Oncology* 2006, **29**(1):66-70.

18. Gupta D, Shukla P, Bisht S S, Aggarwal A, Dhawan A, Pant M C, et al: **Comparitive study of efficacy, tolerability of four field box technique vs. two field anterior posterior technique in locally advanced carcinoma cervix--a prospective analysis**. *Cancer Biology & Therapy* 2009, **8**(9):759-764.

19. Halperin E C, Gaspar L, George S, Darr D, Pinnell S: **A double-blind, randomised, prospective trial to evaluate topical vitamin C solution for the prevention of radiation dermatitis. CNS Cancer Consortium**. *International Journal of Radiation Oncology, Biology, Physics* 1993, **26**(3):413-416.

20. Harsolia A, Kestin L, Grills I, Wallace M, Jolly S, Jones C, et al: **Intensity-modulated radiotherapy results in significant decrease in clinical toxicities compared with conventional wedge-based breast radiotherapy**. *International Journal of Radiation Oncology, Biology, Physics* 2007, **68**(5):1375-1380.

21. Herst P M, Diggelmann K V, Zytkovicz A E, Tuaine J M, Bennett N C, Kelly L E: **Title not available**. In: *International Journal of Radiation Oncology Biology Physics.* San Diego, CA United States; 2010.

22. Hu Y R, Wu C Q, Liu Y J, Wang Y, Li X, Zhong H, et al: **[Clinical observation on effect of shenqi fanghou recipe in preventing and treating radiation injury in patients with head and neck tumor]. [Chinese]**. *Chinese Journal of Integrated Traditional & Western Medicine* 2005, **25**(7):623-625.

23. Korabek BJ: **Comparison of Gentian Violet Application and moisture vapour permeable dressings for the management of open skin lesions secondary to radiation therapy for head and neck cancer**. In: *Thesis.* vol. Master of Science in Nursing. British Columbia: University of Bristish Columbia; 1994.

24. Kouvaris J R, Kouloulias V E, Plataniotis G A, Balafouta E J, Vlahos L J: **Dermatitis during radiation for vulvar carcinoma: prevention and treatment with granulocyte-macrophage colony-stimulating factor impregnated gauze**. *Wound Repair and Regeneration* 2001, **9**(3):187-193.

25. Kouvaris J, Kouloulias V, Kokakis J, Matsopoulos G, Myrsini B, Vlahos L: **The cytoprotective effect of amifostine in acute radiation dermatitis: a retrospective analysis**. *European Journal of Dermatology* 2002, **12**(5):458-462.

26. Livi L, Buonamici F B, Simontacchi G, Scotti V, Fambrini M, Compagnucci A, et al: **Accelerated partial breast irradiation with IMRT: new technical approach and interim analysis of acute toxicity in a phase III randomised clinical trial**. *International Journal of Radiation Oncology, Biology, Physics* 2010, **77**(2):509-515.

27. Løkkevik E, Skovlund E, Reitan J B, Hannisdal E, Tanum G: **Skin treatment with bepanthen cream versus no cream during radiotherapy--a randomised controlled trial**. *Acta Oncologica (Stockholm, Sweden)* 1996, **35**(8):1021-1026.

28. Macmillan M S, Wells M, MacBride S, Raab G M, Munro A, MacDougall H: **Randomized comparison of dry dressings versus hydrogel in management of radiation-induced moist desquamation**. *International Journal of Radiation Oncology, Biology, Physics* 2007, **68**(3):864-872.

29. Maiche A G, Gröhn P, Mäki-Hokkonen H: **Effect of chamomile cream and almond ointment on acute radiation skin reaction**. *Acta oncologica (Stockholm, Sweden)* 1991, **30**(3):395-396.

30. Mak S S S, Molassiotis A, Wan W, Lee I Y M, Chan E S J: **The effects of hydrocolloid dressing and gentian violet on radiation-induced moist desquamation wound healing**. *Cancer Nursing* 2000, **23**(3):220-229.

31. Matceyevsky D, Hahoshen N Y, Vexler A, Noam A, Khafif A, Ben-Yosef R: **Assessing the effectiveness of Dead Sea products as prophylactic agents for acute radiochemotherapy-induced skin and mucosal toxicity in patients with head and neck cancers: a phase 2 study**. *The Israel Medical Association Journal* 2007, **9**(6):439-442.

32. Merchant T E, Kiehna E N, Kun L E, Mulhern R K, Li C, Xiong X, et al: **Phase II trial of conformal radiation therapy for pediatric patients with craniopharyngioma and correlation of surgical factors and radiation dosimetry with change in cognitive function**. *Journal of Neurosurgery* 2006, **104**(2 Suppl):94-102.

33. Miko Enomoto T, Johnson T, Peterson N, Homer L, Walts D, Johnson N: **Combination glutathione and anthocyanins as an alternative for skin care during external-beam radiation**. *American Journal of Surgery* 2005, **189**(5):627-630; discussion 630-621.

34. Mirri M, Soldini P, Pichinelli F, Palloni T, Fabretti F, Ciabattani A: **Topic treatment with Neoviderm cream to prevent and reduce skin toxicity in patients receiving radiotherapy for breast cancer**. In: *TUMORI XIX AIRO 2009 (Conference Abstract).* Bologna; 2009.

35. Momm F, Weissenberger C, Bartelt S, Henke M: **Moist skin care can diminish acute radiation-induced skin toxicity**. *Strahlentherapie und Onkologie* 2003, **179**(10):708-712.

36. Moolenaar M, Poorter R L, Ppg, Lenderink A W, Poortmans P, Egberts A C G: **The effect of honey compared to conventional treatment on healing of radiotherapy-induced skin toxicity in breast cancer patients**. *Acta Oncologica* 2006, **45**(5):623-624.

37. Neben-Wittich M A, Atherton P J, Schwartz D J, Sloan J A, Griffin P C, Deming R L, et al: **Comparison of provider-assessed and patient-reported outcome measures of acute skin toxicity during a Phase III trial of mometasone cream versus placebo during breast radiotherapy: the North Central Cancer Treatment Group (N06C4)**. *International Journal of Radiation Oncology, Biology, Physics* 2011, **81**:397-402.

38. Nyström J, Svensk A C, Lindholm-Sethson B, Geladi P, Larson J, Franzen L: **Comparison of three instrumental methods for the objective evaluation of radiotherapy induced erythema in breast cancer patients and a study of the effect of skin lotions**. *Acta Oncologica* 2007, **46**(7):893-899.

39. Olivotto I A, Weir L M, Kim-Sing C, Bajdik C D, Trevisan C H, Doll C M, et al: **Late cosmetic results of short fractionation for breast conservation**. *Radiotherapy and Oncology* 1996, **41**(1):7-13.

40. Omidvari S, Shafizad A, Razmjou-Ghalaei S, Nasrolahi H, Ahmadloo N, Ansari M, Mosalaei, Mosleh-Shirazi MA, Mohammadianpanah M: **Efficacy of topical honey, topical hydrocortisone 1% and simple washing on healing of radiation-indced dermatitis in breast cancer patients**. *Journal of Isafahan Medical School* 2011, **28**(113):1-8.

41. Patwardhan A, Sharma V, Dinshaw K A: **DUODERM CGF vs Gentian Violet 1% dressing in treatment of radiation induced ulcers**. *World Council of Enterostomal Therapists Journal* 2002, **22**(2):32-38.

42. Pignol J P, Olivotto I, Rakovitch E, Gardner S, Sixel K, Beckham W, et al: **A multicenter randomised trial of breast intensity-modulated radiation therapy to reduce acute radiation dermatitis**. *Journal of Clinical Oncology* 2008, **26**(13):2085-2092.

43. Pignol J P, Olivotto I: **Breast intensity-modulated radiation therapy to reduce radiation dermatitis**. *European journal of Clinical and Medical Oncology* 2010, **2**(2):Not available-Not available.

44. Pinnix C C, Arriaga L, Munsell M F, Perkins G H, Strom E A, Tereffe W, et al: **Title not available**. In: *International Journal of Radiation Oncology Biology Physics.* San Diego, CA United States: International Journal of Radiation Oncology Biology Physics; 2010.

45. Pischnamazzadeh M: **[Prevention of radiation-induced skin reactions in breast cancer]**. *Strahlentherapie* 1983, **159**(1):9-12.

46. Porock D, Kristjanson L: **Skin reactions during radiotherapy for breast cancer: the use and impact of topical agents and dressings**. *European Journal of Cancer Care* 1999, **8**(3):143-153.

47. Robinson M H: **A randomised prospective observer blind trial of E45 cream in the early skin reaction following post-mastectomy chest wall radiotherapy**. *British Journal of Cancer* 1987, **56**(6):878-878.

48. Rudd N, Dempsey S: **Acute skin reaction and psychological benefit of washing with a mild cleansing agent during radiation therapy to the breast or chest wall: a randomised control trial**. *Radiographer* 2002, **49**(2):97-102.

49. Ryan J L, Heckler C E, Pentland A P, Morrow G R: **Potential plasma biomarkers predicting radiation dermatitis in breast cancer patients**. In: *Conference Paper.* vol. Conference: 2010 Annual Meeting of the American Society of Clinical Oncology, ASCO Chicago, IL United States. Conference Start: 20100604 Conference End: 20100608. Conference Publication:; 2010.

50. Röper B, Kaisig D, Auer F, Mergen E, Molls M: **Thêta-Cream versus Bepanthol lotion in breast cancer patients under radiotherapy. A new prophylactic agent in skin care?** *Strahlentherapie und Onkologie* 2004, **180**(5):315-322.

51. Schreck U, Paulsen F, Bamberg M, Budach W: **Intraindividual comparison of two different skin care conceptions in patients undergoing radiotherapy of the head-and-neck region. Creme or powder?** *Strahlentherapie und Onkologie* 2002, **178**(6):321-329.

52. Shoma A, Eldars E, Noman N, Saad M, Elzahaf E, AbdAlla M, Eldin DS, Zayed S, Salaby A, Malek HA: **Pentoxyfylline, and local honey for radiation-induced burn following breast conservative surgery**. *Current Clinical Pharmacology*, **4**:251-256.

53. Shukla P N, Gairola M, Mohanti B K, Rath G K: **Prophylactic beclomethasone spray to the skin during postoperative radiotherapy of carcinoma breast: a prospective randomised study**. *Indian Journal of Cancer* 2006, **43**(4):180-184.

54. Szumacher E, Wighton A, Franssen E, Chow E, Tsao M, Ackerman I, et al: **Phase II study assessing the effectiveness of Biafine cream as a prophylactic agent for radiation-induced acute skin toxicity to the breast in women undergoing radiotherapy with concomitant CMF chemotherapy**. *International Journal of Radiation Oncology, Biology, Physics* 2001, **51**(1):81-86.

55. Vuong T, Franco E, Lehnert S, Lambert C, Portelance L, Nasr E, et al: **Silver leaf nylon dressing to prevent radiation dermatitis in patients undergoing chemotherapy and external beam radiotherapy to the perineum**. *International Journal of Radiation Oncology, Biology, Physics* 2004, **59**(3):809-814.

56. Westbury C, Hines F, Hawkes E, Ashley S, Brada M: **Advice on hair and scalp care during cranial radiotherapy: a prospective randomised trial**. *Radiotherapy and Oncology* 2000, **54**(2):109-116.

57. Wollina U, Christen N, Kostler E, Schorcht J: **On prophylaxis and treatment of radition-induced dermatitis and mucositis**. *Zeitschrift fur Hautkrankheiten* 2002, **77**(9):418-423.

58. Zhou C H, Fan C H, Cheng H J, Wang X Y: **[Effects of Shi Ren Shao Shang plaster ( a moistening plaster for healing burning injury) in treatment for acute irradiation dermatitis]**. *Chinese Journal of Radiation Oncology* 1993, **2**(4):262-262.
