# Supplementary material for: Prevention and treatment of acute radiation-induced skin reactions: a systematic review and meta-analysis of randomized controlled trials
Source: BMC Cancer. 2014 Jan 31;14:53. doi: 10.1186/1471-2407-14-53 (PMC3909507; doi:10.1186/1471-2407-14-53)
Supplement: Additional file 3 — Data extraction sheet. [file 1471-2407-14-53-S3.doc]

**Additional File 3- Data Extraction Sheet**

**Eligibility of study for review: (consider PICO)**

- Participants

- Intervention

- Comparison

- Outcomes

**Setting:** (where was this study conducted?)

**Start and end date** (including follow-up times)

**Funding source** (including role of funders if known)

**Conflict of interest**

**Participants and Included criteria and exclusion criteria**

**Ethics approval:**

**Consent:**

**What were the primary/ secondary outcome measures:**

**Who assessed the primary/ secondary outcome measures:**

**When were these prim ray/ secondary outcome measures assessed:**

**Were validated outcome measures used?** (Yes/No, for which measures)

**Participants characteristics** (age/gender)

**Loss to** follow-up **with reasons:** (number and %)

**Final number of participants:**

**Participants excluded from the study:**

**Describe the interventions each group received**

- Please specify supplier and trade name if relevant

Were instructions given to patients adequate? Give details

**Results:**

Dichotomous/ Categorical outcomes (number of events/ total number of each group) Details if outcome only described in text

Continuous data (mean and standard deviations, number analysed) (provide other information is appropriate, e.g. SE/ 95% CI, p values) Details if outcome only described in text

**Indicate below if results were estimated from graphs etc; or calculated using a formula (this should be stated in the review and the formula given).**

**In general, if results were not reported in the paper, but were obtained from another source, this should be made clear in the review.**

**Questions to ask authors/ responses**
