# Supplementary material for: Prevention and treatment of acute radiation-induced skin reactions: a systematic review and meta-analysis of randomized controlled trials
Source: BMC Cancer. 2014 Jan 31;14:53. doi: 10.1186/1471-2407-14-53 (PMC3909507; doi:10.1186/1471-2407-14-53)
Supplement: Additional file 4 — Characteristics of included studies. [file 1471-2407-14-53-S4.doc]

**Additional File 4- Characteristics of included studies**

Aquino-Parsons 2010

| **Methods** | **Design:** Randomised controlled trial  **Setting:** Department of Radiation Oncology, Vancouver Cancer Centre, British Columbia Cancer Agency, Vancouver, British Columbia, Canada  **Date of study:** Unclear |
| --- | --- |
| **Participants** | **Inclusion criteria:**  - Women with in situ or invasive breast cancer after breast-conserving surgery and scheduled to receive adjuvant whole breast radiation treatment who were deemed to be at high risk of developing inframammary radiodermatitis from breast size or shape  - Included cup size D or greater, cup size C with 1cm or greater skin fold in the treatment position or any cup size with 2 cm or greater skin fold in the treatment position  - Patients undergoing an electron boost to the tumour bed were eligible if the surgical scar did not extend into inferior hemisphere of the breast    **Exclusion criteria:**  - Any known allergy to silver, significant connective tissue disease, known radiation hypersensitivity syndrome, or if there had been a significant wound complication, either infection or dehiscence.  - Patients were not eligible if the patients stated before consenting that they were not willing or able to return to the clinic for this additional study visits. |
| **Interventions** | **Intervention:**  A: Silver Leaf Nylon Dressing (SLND)  Patients were supplied with dressing, sterile water, a cotton brassiere in the correct size, gauze, hypo-allergic tape, a printed instruction sheet, and were taught by study radiation therapists how to wear the SLND before day 6 of radiation treatment. The patients were then seen on their sixth fraction of radiation treatment, the day they would start wearing the SLND, to ensure their understanding and compliance. Sterile water moistened the dressing before application into the inframammary fold with gauze to cover it. This was held in place by the cotton brassiere and was to be worn 24 hours per day, but removed for each radiation treatment and bathing. It was worn from the day of the 6^th^ fraction of radiation treatment until 14 days after completion of whole breast radiation treatment. In addition, women in this arm also received the standard skin care instructions.  **Control intervention:**  B: Standard skin care recommendations  - Avoidance of skin irritants, promotion of cleanliness (with non-alkaline, unscented soap and water, and patting dry with a soft towel), and maintenance of skin hydration with twice-daily application of a moisturising cream. Patients chose which brand of moisturising cream to use. The development of pruritus or brisk erythema was managed with topical steroids. Moist desquamation was treated with twice-daily saline compresses and the use of hydrogel or silver sulfadiazine cream as required. |
| **Outcomes** | **Primary outcomes of the trial:**  - Presence of moist-desquamation (objective assessments using photographs)  - The maximum area of moist desquamation (objective assessments using photographs)  - The degree of erythema (objective assessments using photographs)  - RTOG skin toxicity scores  (objective assessments using photographs)  - Pain (Patients rating using a 10 cm visual analogue scale)  - Itching (Patients rating using a 10 cm visual analogue scale)  - Burning sensation (Patients rating using a 10 cm visual analogue scale)  **Assessment time points:**  - one week before the last whole breast radiation treatment, on the last day of treatment and one week subsequent |
| **Notes** |  |

Aygenc 2004

| **Methods** | **Design:** Randomised controlled trial  **Setting:** Not stated (Turkey)  **Date of study:** October 1999 - October 2001 |
| --- | --- |
| **Participants** | **Inclusion criteria:**  Patients receiving postoperative RT for squamous cell carcinoma (SCC) of the head and neck |
| **Interventions** | **Intervention:**  A: Patients were given at a dose of 400 mg of Pentoxifyline 3 times a day orally starting from the first day of irradiation until 2 weeks after the completion of RT. The dose was reduced to 400 mg twice daily in patients experiencing side effects  **Control intervention:**  B: Usual care not defined |
| **Outcomes** | **Primary outcomes of the trial:**  - Scoring system was developed by Baker-Liegh & Dion)  - Uncertain if this is a validated scale  - Early RISR: during treatment and 8 weeks after  - Late RISR: Eight weeks plus  **Secondary outcomes of the trial:**  - Adverse effects (defined as "unable to tolerate drug at prescribed level)" |
| **Notes** | - Author used available case analysis, not intention to treat analysis |

Bairati 2005

| **Methods** | **Design:** Double blind, placebo-controlled randomised controlled trial  **Setting:** Five Radiation therapy centres in Quebec, Canada  **Date of study:** October 1, 1994 – June 6, 2000 |
| --- | --- |
| **Participants** | **Inclusion criteria:**  Patients with stage I or II Head and neck squamous cell carcinoma of the head and neck area. These head and neck areas included skin, mucosa, ear, salivary glands, pharynx and oesophagus and larynx. |
| **Interventions** | **Intervention:**  A: Received α-tocopherol and β-carotene supplementation (first 79 randomised to this group). Then stopped β-carotene (went to 1 supplement) due to a trial suggesting increased risk of lung cancer. This change was recommended and approved by the local ethics approval body. Daily supplementation during radiation therapy and for 3 years after. The supplementation began on 1^st^ day of RT. Capsules supplied by Roche vitamins Inc (Parsippany, NJ)  **Control Intervention:**  B: Corresponding placebos during radiation therapy and for 3 years |
| **Outcomes** | **Primary outcomes of the trial:**  - trial authors only reported as overall statistic only for overall Acute adverse effects of radiation therapy (not skin specific) (OR) (Investigated was contacted with no reply on the date of publication of this review)  - Mean HNRQ score for skin (range 1-7; where 7 = no symptoms)  **Secondary outcomes of the trial:**  Quality of life- measured at the end of treatment and one month later using assessed by the Quality of Life Questionnaire C30 and the  HNC-specific quality-of-life questionnaire  **Adverse effects:** Included with other outcomes as an overall measure |
| **Notes** |  |

Bennett 2009

| **Methods** | **Design:** Randomised controlled trial  **Setting:** Bristol Haematology and Oncology Centre (Bristol, UK)  **Date of study:** May 2004 and February 2005 |
| --- | --- |
| **Participants** | **Inclusion criteria:**  All women receiving post-operative radical radiation treatment for cancer of the breast (including ductal carcinoma in situ) with or without treatment to the axilla. |
| **Interventions** | **Intervention:**  A: Specific non-metallic deodorant. Patients in the deodorant group were given instructions for its use.  **Control intervention:**  B: No deodorant |
| **Outcomes** | **Primary outcome of the trial:**  Radiation Therapy Oncology Group (RTOG) scale |
| **Notes** | - The researcher was the only person involved in the design, conduct and assessment of this study |

Campbell 1992

| **Methods** | **Design:** Randomised controlled trial  **Setting:** Mersey Regional Centre for Radiotherapy and Oncology, (Merseyside  UK)  **Date of study:** Not stated |
| --- | --- |
| **Participants** | **Inclusion:**  Patients were all receiving adjuvant postoperative radiation treatment following treatment of a breast carcinoma by either local excision or mastectomy |
| **Interventions** | **Intervention:**  A: Patients were told not to wash or wet the treated skin surface at all  **Control intervention:**  B: (Water only) Patients were told “You may wet the treated skin surface in a warm (not hot) shower or by immersion in a warm bath.'; 'Do not soak in the bath and do not use soap.'; 'Do not rub or scrub the treated skin.'; 'Do not rub the treated skin dry with a rough towel- either drip dry or pat it dry with a towel.'  C: (Soap and water) Patients were told “You may wash the treated skin surface in a warm (not hot) shower or by immersion in a warm bath. Use baby soap or simple soap. Do not use shower gels, bubble baths or body lotions.'; 'Do not soak in the bath and do not scrub the treated skin.'; 'Try to avoid the use of a flannel or sponge.'; 'Do not rub the treated skin dry with a rough towel - either drip dry or pat it dry with a towel.'  **Co-intervention:**  Patients in all groups received the same management advice regarding other forms of skin care |
| **Outcomes** | **Primary outcome of the trial:**  EORTC/RTOG acute skin reaction scoring system. |
| **Notes** | Unable to contact author to obtain further information |

Dale 2001

| **Methods** | **Design:** Randomised Controlled Trial  **Setting:** Nargis Dutt Memorial Cancer Hospital, (Maharashtra, India)  **Date of study:** October 1996 – September 1997 |
| --- | --- |
| **Participants** | **Inclusion criteria:**  Patients with locally advanced, biopsy confirmed carcinoma of the uterine cervix stages IIA, IIB, or IIIB |
| **Interventions** | **Intervention:**  A: One tablet of the test drug Wobe-Mugos E contains 100mg papain, 40 mg trypsin, & 40mg chymotrypsin.  Patients were given 3 tablets 4 times a day beginning 7 days before radiation started.  **Control intervention:**  B: No medication |
| **Outcomes** | **Primary Outcomes:**  - "RTOG/EORTC criteria" was used.  - All patients were monitored weekly for 5 weeks, and followed up at intervals of 6 weeks, and 4 months after completion of radiation therapy. |
| **Notes** | Mucos Pharma (manufacturer of the intervention treatment) assisted with manuscript writing. Sources for the study funding not disclosed. |

Delaney 1997

| **Methods** | **Design:** Double Blind, Placebo controlled, Randomised Controlled Trial  **Setting:** Liverpool hospital, NSW (Australia)  **Date of study:** Jan 1992 – Jan 1994 |
| --- | --- |
| **Participants** | **Inclusion criteria:**  Patients receiving radiation therapy to their head and neck cancer, breast cancer and other malignancies. Patients were eligible if they had developed a measurable area of moist desquamation (Radiation Therapy Oncology Group (RTOG) grade 3 skin reaction) in the irradiated area before completion of radiation treatment, and were agreeable to continue follow-up until resolution of moist desquamation.  **Exclusion criteria:**  Patients were not eligible if they had developed desquamation only after completion of the radiation treatment or had been taking non-steroidal anti-inflammatory medicine. |
| **Interventions** | **Intervention:**  A: Sucralfate  Sucralfate was mixed with sorbolene as10% w/w (i.e. 50 g of sucralfate crushed in 500 g of sorbolene).  Topical application of the allotted treatment was commenced on the date of entry to the trial. A liberal amount of cream was applied to the irradiated area three times daily, with a salt water bath preceding each application.  **Control intervention:**  B: Sorbolene cream  Topical application of the allotted treatment was commenced on the date of entry to the trial. A liberal amount of cream was applied to the irradiated area three times daily, with a salt water bath preceding each application. |
| **Outcomes** | **Primary outcome of the trial:**  - Time to heal from the date of randomisation  **Secondary outcome of the trial:**  - Pain relief  - Adverse effects  Assessment: Assessment was conducted by patients and the investigator. Patients were required to fill out a daily patient assessment form while participating in the trial. This involved providing five responses. The level of discomfort, change (improvement/ deterioration) in discomfort and degree of pain when applying the cream were assessed by linear analogue self-assessments (LASA). Patients were also asked whether they had noticed any adverse effects from the cream and for the time taken for pain improvement following application of the paste.  Additionally, patients were assessed twice weekly by the investigator. At each assessment, the area of moist desquamation was measured and photographed. |
| **Notes** | **Quote: "**The present study was financially supported by the Boots Company (Australia) Pty Ltd, the Australian suppliers of sucralfate. All decisions regarding the design, conduct and analysis of the trial were made independently of the company." |

Elliott 2006

| **Methods** | **Design:** Randomised controlled trial  **Setting:** 51 Radiation treatment centres across North America  **Date of study:** October 2000 to April 2002 |
| --- | --- |
| **Participants** | **Inclusion criteria:**  Patients with biopsy proven stage III or IV cancer of the oral cavity, oropharynx hypopharynx or larynx |
| **Interventions** | **Intervention:**  A: (Prophylactic trolamine)  Instructed to apply trolamine 3 times daily (at 4-hourly intervals) from the first day of treatment until 2 weeks after treatment.  B: (Interventional trolamine)  Instructed to apply trolamine 3 times daily (at 4-hourly intervals) from the time their skin became itchy, bothersome or reddened until 2 weeks after treatment  **Co-intervention for A and B:**  Those in both trolamine groups were advised to wash the area regularly with warm water and mild soap to prevent build up. Bolus was permitted at the discretion of the treating physician  **Control intervention: (Institutional preference)**  C: Fourteen different institutional preferences were recorded at study registration. |
| **Outcomes** | NCI-CTC for toxicity and Spitzer QOL index and specifically designed HNRQ |
| **Notes** | - Medix Pharmaceuticals supplied all the trolamine emulsion.  - The author was unable to be contacted. An auto-reply indicated that the first author has now retired. The co-authors did not reply to correspondence. |

Fife 2010

| **Methods** | **Design:** Double-blind, randomised controlled trial  **Setting:** The Department of Radiation Oncology at the University of California, Irvine Medical Center(Orange, CA)  **Date of study:** Not stated |
| --- | --- |
| **Participants** | **Inclusion criteria:**  - patients had to be aged 18 and older and have clinically diagnosed breast cancer that would be treated with RT. All patients had undergone a prior lumpectomy or mastectomy.  **Exclusion criteria:**  Patients who were pregnant or lactating were excluded from the study. |
| **Interventions** | **Intervention:**  A: LED treatment  Patients in the treatment group received LED treatments immediately before and after each radiation session. “Each LED treatment was administered using the GentleWaves Select 590-nm high-energy LED array with the panel being placed within 2 cm of the patient’s skin. Each treatment lasted 35 seconds, using the same specific sequence of pulses used by DeLand and colleagues and in other studies, in which the pulses are 250 ms on and 100 ms off for 100 pulses. Upon completion of the RT course, seven additional daily treatments were given over the next 2 weeks with the goal of preventing the delayed reaction seen in RT.”  **Control intervention:**  B: Sham treatment  Patients in the control group had sham treatments, in which the machine was placed on the skin at the same times in the same manner for the same duration of 35 seconds, but the button was not pressed to deliver the light. Patients in both groups had towels or eye shields placed over their eyes to blind them as to whether the LED device was administering light treatments. |
| **Outcomes** | **Primary outcomes of the trial:**  Skin reactions: A dermatologist blinded to study group (KMK) graded standardized photographs of the week five visit according to the National Cancer Institute (NCI) 5-point scale for grading skin reactions    **Assessment time points for primary outcomes:**  - baseline, weekly during ongoing RT, at the completion of RT, and 2 and 6 weeks after the completion of RT.    **Secondary outcomes of the trial:**  patients assess the convenience, discomfort, and overall satisfaction with their treatment,  as well as their assessment of the cosmetic appearance of the skin on a 6-point (0–5) scale.    **Assessment time points for secondary outcomes:**  - Weekly during treatment and at the end of the entire treatment course |
| **Notes** |  |

Fisher 2000

| **Methods** | **Design:** Randomised controlled trial  **Setting:** Radiation Oncology centre, Oakwood Hospital and Medical centre (MI, USA)  **Date of study:** Between February 1, 1998 and May 1, 1998 |
| --- | --- |
| **Participants** | **Inclusion criteria:**  Patients with confirmed diagnosis of breast cancer and a planned course of radiation  therapy to the breast for a minimum dose of 50 cGy, including the boost. |
| **Interventions** | **Intervention:**  A (Biafine)  Patients were instructed to begin applying assigned product (biafine) following their first radiation treatment and to do this three times a day until 2 weeks following completion of their radiation treatments. The assigned product was not to be applied within 4 h of their daily radiation session.  **Control intervention:**  B (Institutional preference)  Patients were instructed to begin applying assigned product following their first radiation treatment and to do this three times a day until 2 weeks following completion of their radiation treatments. The assigned product was not to be applied within 4 h of their daily radiation session.  **Co-intervention:**  If Grade 3 or 4 toxicity occurred, additional treatment and continuation of study product was at the physician’s discretion. |
| **Outcomes** | **Primary outcomes of the trial:**  Skin dermatitis was scored weekly utilizing both the RTOG Acute Toxicity Scale and the Oncology Nursing Society (ONS) Radiation Therapy Care Record for Breast. The ONS scale was used, but not reported in this article because it is an invalidated scale. QOL assessed using the Spitzer quality-of-life questionnaires |
| **Notes** |  |

Garibaldi 2009

| **Methods** | **Design:** Randomised controlled trial  **Setting:** Institute for Cancer Research & Treatment, (Turin, Italy)  **Date of study:** 2007 |
| --- | --- |
| **Participants** | Patients with breast cancer or head and neck cancer  **Inclusion criteria:**  - Patients with breast cancer operated on conservatively and requiring postoperative radiation treatment.  - patients with head and neck cancer requiring radiation treatment with radical intent  - Karnofsky performance status 90-100  - patients with negative history for allergy.  **Exclusion criteria:**  - Patients with history of cutaneous hypersensitivity to topical substances  - Patients with positive history for allergy  - Patients with connective tissue diseases  - Patients with history of psychological problems that could reduce compliance to treatment protocol |
| **Interventions** | **Intervention:**  A: Restitutio Restructuring Cream (RRC) Formula A (by Ganassini Institute)    Patients self-administered the cream, in quantities of about 3 grams per application from the first day of radiation treatment until the 10^th^ day after the end of radiation treatment. Cream was applied twice a day, three hours before and immediately after radiation.  **Control intervention:**  B: Restitutio Restructuring Cream (RRC) Formula B    Patients self-administered the cream, in quantities of about 3 grams per application from the first day of radiation treatment until the 10^th^ day after the end of radiation treatment. Cream was applied twice a day, three hours before and immediately after radiation. |
| **Outcomes** | **Primary outcomes of the trial:**  Skin dermatitis was scored weekly utilizing the RTOG Acute Toxicity Scale. |
| **Notes** |  |

Gee 2000

| **Methods** | **Design:** Randomised controlled trial.  **Setting:** Catterbridge Centre for Oncology, Merseyside, UK  **Date of study:** October 1996 – March 1997 |
| --- | --- |
| **Participants** | **Inclusion Criteria:**  Patients receiving standard radiation treatment for breast cancer, at 45 Gy in 20 treatments, given over 28 days, using 5MV photons.  No restriction on age  Could speak English  Patients receiving treatment to both of their breast and axilla as well as those that were having breast only treatment were included.  People receiving cytotoxic chemotherapy were included. |
| **Interventions** | **Intervention:**  A: Deodorant group  - Received the conventional skin care leaflets and verbal instructions, usually given by the radiographers. They were also given non-metallic-deodorant  **Control intervention:**  B: No deodorant group  Received the leaflet and verbal instructions but no-deodorants. |
| **Outcomes** | **Primary outcomes of the trial:**  Weekly assessment by patients  - Rotterdam Symptom Checklist (RSCL)  - Itching, tightness, burning, pain:  0- none, 1- mild, 2-moderate, 3- severe    Weekly assessment by a consultant and three registrars  - erythema:  0- none, 1- mild, 2-moderate, 3- severe  - Desquamation  Dryness of skin  Moderate flaking  Severe flaking or patchy moist desquamation |
| **Notes** | **Comment:** No other potential threats to validity were identified |

Glees 1979

| **Methods** | **Design:** Double blind, randomised controlled trial  **Setting:** The Royal Marsden Hospital, Fulham Road, (London, England)  **Date of study:** Not stated |
| --- | --- |
| **Participants** | **Inclusion criteria:**  All patients with a diagnosis of carcinoma of the breast requiring radical radiation treatment to the breast or chest wall if they had a mastectomy, and gland areas, were asked to participate. |
| **Interventions** | **Intervention:**  A: (Hydrocortisone)  Patients were given a 30g tube of 1% hydrocortisone cream the tubes bearing the trial number of each particular patient.  - All patients were given a daily record card on which they were to record their subjective impression of the skin reaction. These were filled in from the start of radiation therapy so that the time taken for the onset of dermatitis could be recorded.  **Control intervention:**  B: (Clobetasone)  Patients were given a 30g tube of 0.05% clobetasone butyrate (Eumovate) cream, the tubes bearing the trial number of each particular patient.  - All patients were given a daily record card on which they were to record their subjective impression of the skin reaction. These were filled in from the start of radiation therapy so that the time taken for the onset of dermatitis could be recorded. |
| **Outcomes** | - The symptomatic relief of dermatitis as experienced and noted down by the patient.  - The amount of steroid preparation used  - The clinical response of the patients i.e. nature of skin reaction and the dosage achieved, as noted by the radiotherapist.  - The number of length of the 'rest periods' from treatment required by each group.  - The patient's assessment of onset of specific symptoms tabulated in order of frequency severity and time of onset (e.g. soreness, burning or tingling, itching, redness, peeling).  - The author's assessment as to whether 'the cream' was of benefit or no benefit to each patient at the end of the course of radiation treatment prior to breaking the code. |
| **Notes** | The third author is a “Medical Associate, Medical Department, Glaxo Laboratories Limited, Greenford, Middlesex. However, it is unclear if Glaxo was the manufacturer of either product." |

Gollins 2008

| **Methods** | **Design:** Randomised controlled trial  **Setting:** Regional cancer centre in Wales  **Date of study:** Between February 1996 and May 1997 |
| --- | --- |
| **Participants** | **Inclusion criteria:**  Patients at one regional cancer centre receiving either adjuvant radiation treatment to the chest wall following mastectomy, adjuvant radiation treatment to the breast following wide local excision of a breast carcinoma, or radiation treatment to any region of the head and neck who then developed an area(s) of moist desquamation within the radiation treatment field were recruited into the study. The patient had to be judged capable of complying with instructions for dressing application, and have an expected life expectancy of more than three months.  **Exclusion criteria:** Patients were excluded if there was any dehiscence of a previous surgical incision within the desquamated area, the affected area had been treated previously with a course of radiation treatment, there was still a macroscopic tumour in the area at the start of radiation treatment, or it showed clinical signs of infection. Patients only received chemotherapy after their desquamation had fully healed. |
| **Interventions** | **Intervention:**  A: Hydrogel  "hydrogel dressing (2nd Skin, Spenco). '2nd Skin' is a sterile, transparent, cross-linked polyethylene oxide hydrogel consisting of 96% water and 4% polyethylene oxide.  Patients were given the following instructions:  - Do not powder the affected area  - Gently cleanse the raw skin by squeezing a cotton wool ball that has been soaked in cool boiled tap water over it. Try not to touch the raw area of skin and do not rub your skin as this will make it sorer  - Peel the two sides of the 2nd Skin pack apart.  Inside you will find the sheet of gel, which is protected on one side by blue cellophane and on the other by clear cellophane  - Holding the sheet flat, remove the blue cellophane and gently place the gel on the sore area  - You may find your clothing is sufficient to keep the gel in place, but if not it can be covered lightly with a padded dressing and some tape. (Padded dressings used over 2nd Skin on the breast can often be held in place with a bra.) The radiographers will show you how to do this and it is important not to put tape on skin in the radiation treatment field  - You can leave the gel on for up to 12 hours and it will remain moist, but it should not be left on any longer. If it has begun to dry, the gel should be gently wetted before removal with cool sterile water from a cotton wool ball  - When you remove the gel, you may notice a yellow mark on your skin. This is normal and does not mean your skin has become infected. Gently cleanse the skin by squeezing a cotton wool ball soaked in sterile water over it, or irrigating it with sterile water from a syringe. Apply another 2nd Skin, as above."  *Hydrogel group cleansed the area with  Sterile water before applying product.  **Control intervention:**  B: Gentian violet  0.5% aqueous GV was used, and patients were given the following instructions:  - Do not powder the affected area  - Wear disposable gloves  - Use cotton wool balls to apply the GV to the raw area of skin by gently dabbing it rather than rubbing as this will make your skin more ulcerated  - When there is a good first coat of GV, dry with a cold air-blowing hair dryer or fan  - Gentian violet should be applied in this way several times until there is a good crust over the area  - Apply GV several times a day to keep a protective crust over the area. During the first few days you will probably have to apply the GV approximately four times a day, but as healing gradually takes place you will be able to use it less often during the day  - Do not remove the previous application of GV  - The area should be kept open as much as possible, but if staining of clothes is a problem it can be covered with a dry dressing as long as there is a good crust of gentian over the raw area. The radiographers will show you how to do this and it is important that, if you use tape, not to put it on skin in the radiation treatment field.  *GV group dried area with a cool hair dryer after applying GV |
| **Outcomes** | **Primary outcomes for the trial:**  - Time to complete healing of the desquamated area  **Secondary outcomes for the trial:**  - 'Area under the curve' of the plot of the median area of moist desquamation against time |
| **Notes** | The study was funded by internal Velindre NHS Trust hospital funds. |

Gosselin 2010

| **Methods** | **Design:** Randomised control, placebo-controlled, randomised controlled trial  **Setting:** Department of Radiation Oncology at Duke University Hospital. (USA)  **Date of study:** 2002-2006 |
| --- | --- |
| **Participants** | **Inclusion criteria:**  Women with a diagnosis of breast cancer who were to receive whole-breast radiation therapy.  Female, diagnosis of breast cancer, older than 18 years, Karnofsky performance status of 80 and higher and the ability to read and write English.  **Exclusion criteria:**  Patients with skin lesions on either breasts, are pregnant, mastectomy, concurrent chemotherapy, concurrent hyperthermia, prior radiation to the same breast and history of allergic reaction to the products used in the study were excluded. |
| **Interventions** | **Intervention:**  A: Aquaphor (ointment)  B: RadiaCare TM (Carrington Laboratories,Inc.) (gel)  **Control interventions:**  C: Placebo (sterile water mist)  Patient instructions:  Products were given to participants in a brown bag along with instructions for application.  Participants were able to bathe.  Patients were instructed to start the product on the first day of treatment and apply it twice a day (morning and night) every day of the week until treatment was complete, document the application in the journal, wash hands before and after product use, and avoid applying any other skin care product to the breast or applying the study product four hours before treatment. If radiation therapy was received in the morning, patients were instructed to apply the product after treatment. Participants were provided additional product as needed throughout their course of radiation |
| **Outcomes** | **Primary outcomes of the trial:**  Clinician assessment using the RTOG criteria  **Secondary outcomes of the trial:**  Patient's rating of satisfaction and ease to use |
| **Notes** | - Products were supplied by the investigational pharmacy (not clear which products the pharmacy produced or who the pharmacy were).  - The conduct of this study was supported by the Oncology Nursing Society Foundation through an unrestricted grant from Aventis Pharmaceuticals. |

Gujral 2001

| **Methods** | **Design:** Randomised controlled trial  **Setting:** The SGOT Cancer Hospital, Indore, (Madhya Predesh, India); AH Regional Cancer Centre, Cuttack, (Orissa, India)  **Date of study:** Aug 1996 to April 1997 |
| --- | --- |
| **Participants** | **Inclusion criteria:**  - Patients aged 18-65 years  - Biopsy-proven squamous cell carcinoma, Head and Neck cancer  - Previous chemotherapy was allowed, but no prior radiation therapy  **Exclusion criteria:**  - Patients with distant metastasis  - Karnovsky Index <70 or altered haematological or biochemical parameters |
| **Interventions** | **Intervention:**  A:  Usual radiation therapy plus enzyme therapy (Wobe-Mugos E, Pharma, Geretsried, Germany), containing papain (100mg), trypsin (40mg) and chymotrypsin (40mg).  Three tablets were given three times/day from three days prior to commencing radiation therapy until five days after completion of therapy.  **Control intervention:**  B:  Usual radiation therapy. No placebo treatment.  **Co-intervention:**  Before the start of radiation therapy, all the patients were advised to maintain high oral hygiene after a complete dental check-up. No prophylactic treatment aimed at mucositis was used. Non-steroidal anti-inflammatory drugs such as diclofenac and ibuprofen were prescribed only as rescue medication, when patients reported moderate pain and patchy mucositis. In cases of severe pain and confluent mucositis, morphine analogues such as dextropropoxyphen and steroids were used. |
| **Outcomes** | **Primary outcomes of the trial:**  - Maximum severity of skin reactions during a planned period of radiation therapy  - Time until the maximum toxicity scores  - Overall sum scores  - Skin reactions were graded using RTOG/EORTC  Patients were first evaluated for baseline data, then, evaluation was continued at weekly intervals for 6 to 8 weeks covering the period of radiation therapy, and for another 5 to 6 months after the end of radiation therapy.  The scoring was done by the same investigator |
| **Notes** | This could be the same trial as reported by Kaul 1999. Attempts were made to contact the authors to verify this. However, no replies have been received on the date of publication of this interview. |

Halnan 1962

| **Methods** | **Design:** Randomised controlled trial  **Setting:** The Christie Hospital and Holt Radium Institute, (Manchester, UK)  **Date of study:** Not stated |
| --- | --- |
| **Participants** | **Inclusion criteria:**  - New untreated basal cell cancer on the face for treatment by a 3 cm diameter circular field of superficial X rays as below  - Ability and willingness to attend as frequently as required for follow-up  **Exclusion criteria**  Lesions close to the eye |
| **Interventions** | **Intervention:**  A: Steroid and antibiotic ointment  Ointment containing  0.5 % Prednisolone Diodium phosphate and 0.5% Neomycin sulphate in a water miscible polyethylene glycol type base. 15g tube supplied, Patients were asked to apply sparingly twice daily  B: antibiotic control ointment  Identical to group A’s cream except for the absence of Prednisolone. 15g tube supplied, Patients were asked to apply sparingly twice daily  **Control intervention:**  C: No ointment |
| **Outcomes** | **Primary outcome of the trial:**  Skin reaction, no further information is given |
| **Notes** |  |

Heggie 2002

| **Methods** | **Design:** Randomised controlled trial  **Setting:** Queensland Radium Institute (the Mater Centre and the Royal Brisbane Hospital Centre) (Brisbane, Australia)  **Date of study:** Not stated |
| --- | --- |
| **Participants** | **Inclusion criteria:**  18 years of age who had undergone lumpectomy or partial mastectomy for breast cancer, and who then received postoperative radiation therapy using tangential field, with or without boost to the tumour bed. Patients could have concurrent chemotherapy.    **Exclusion criteria:**  Those requiring nodal radiation therapy |
| **Interventions** | **Intervention:**  A: Aloe Vera  Experimental arm using topical 98% aloe Vera gel on the irradiated breast to be applied three times daily throughout and for 2 weeks after radiation therapy was completed.  **Control intervention:**  B: Aqueous cream  A control arm using topical aqueous cream on the irradiated breast to be applied three times daily throughout and for 2 weeks after radiation therapy was completed.  **Co-intervention:**  Using only mild baby soap on the treatment site, airing skin twice daily, and wearing loose cotton clothing next to treated skin. |
| **Outcomes** | **Primary outcomes of the trial:**  Skin reactions: Research nurses performed skin assessments each week using the Morbidity Rating Scale. |
| **Notes** |  |

Jensen 2011

| **Methods** | **Design:** Randomised Controlled Trial  **Setting:** Department of Therapeutic Radiology, university Hospitals of Schleswig-Holstein, University of Kiel, (Germany)  **Date of study:** Not stated |
| --- | --- |
| **Participants** | **Inclusion criteria:**  Adult females who had undergone radiation treatment for breast cancer following surgery and were experiencing radiodermatitis with an ONS score of 0–3 |
| **Interventions** | **Intervention:**  A: Local treatment with WO1932 was initiated directly after termination of the radiation therapy and was applied by the patients twice daily over the 6–8 week treatment period.  WO1932 is based on an oil-in-water emulsion with high water content (83%) and is classified as a medical device under German law.  The oil-in-water emulsion contains unsaturated fatty acids among other excipients, in particular a mixture of 9,11- and 9,12-octa- decadienoic acid (linoleic acid).  Sauna, swimming, and the use of any skin care products like creams, bath oils, etc. in the test area were not allowed during the study.  **Control intervention:**  B: The control group remained untreated. |
| **Outcomes** | **Primary outcomes of the trial:**  Biophysical measurements (stratum corneum hydration and transepidermal water loss (TEWL)) and clinical assessments (pruritus and ONS scoring) of both groups were performed at baseline (visit 1, day 1), after 1 week (visit 2, day 8), and after 6–8 weeks of treatment (visit 3).    ONS radiation skin reaction scoring was used to assess disease severity of irradiated skin [24]. Each symptom of erythema and scaling was given a score ranging from 0 (none) to 3 (severe). Pruritus was recorded by the patients in a pruritus diary, according to a visual analogue scale from 1–10 (1 = no pruritus, 10 = severe, intolerable pruritus). This visual analogue scale (length in cm) was transformed to a scale ranging from 0 (no pruritus) to 100 (severe).    Stratum corneum hydration and TEWL were determined at each visit in both lesional and non-lesional skin using the Corneometer® and the Tewameter® TM210 (both Courage & Khazaka, Cologne, Germany) in accordance with the guide- lines of the Standardization Group of the European Contact Dermatitis Society. |
| **Notes** | This work was supported by grants from the Deutsche Forschungsgemeinschaft (SFB415/B2 and SFB617/A7, A21) given to Ehrhardt Proksch, and Dr. August Wolff GmbH & Co. KG, Bielefeld (Germany) given to Ehrhardt Proksch and Jens-Michael Jensen. Ehrhardt Proksch and Theodor May have acted as consultants to Dr. August Wolff GmbH & Co. KG. Christoph Abels and Gunter Lemnitz are employed by Dr. August Wolff GmbH & Co.  Some authors seem to be employees of the Emulsion manufacturer.  - No cross tabulation comparing the demographic and medical characteristics of patients. |

Kirova 2011

| **Methods** | **Design:** Randomised controlled trial  **Setting:** Radiotherapy Oncology department, Institute Curie, (Paris France)  **Date of study:** April 2005 to October 2008 |
| --- | --- |
| **Participants** | **Inclusion criteria:**  Women 18 years and older who are undergoing normo-fractionated loco regional radiation treatment for breast cancer and who presented with grade 1 radio induced dermatitis during or after radiation.  **Exclusion criteria:**  concurrent chemotherapy, severe cognitive disorders and tumoral wound in the irradiated area |
| **Interventions** | **Intervention:**  A: Hyluronic acid cream  100mg tube of Laluset, Genevrier, France.  Apply once a day  Instructed to shower 1-2 times daily so as to have a clear skin a time of radiation.  Both Oral and written instructions given detailing preventative measures  **Control intervention:**  B: Placebo  200 ml tube of Topi cream, Charlieu, France.  Apply once a day  Instructed to shower 1-2 times daily so as to have a clear skin a time of radiation.  Both Oral and written instructions given detailing preventative measures |
| **Outcomes** | **Primary outcomes of the trial:**  - Pain- Visual Analouge Scale  - Quality of life- EORTC QLQ-C30  - Dermatitis was based on the RTOG scale.  - "The cutaneous colorimetric assessment was performed with a chromameter (Minolta CR300) according to the International Commission on Illumination classification. Each colour represented a point defined by its three co-ordinates (L*a*b*). To calculate the intensity of the erythema, the ‘‘a*’’ parameter (red) was used. Two measures were registered: the first on a normal area used as reference (a*), and the second" on the erythema (a*1)" |
| **Notes** | **“**Laboratoire Genevrier France provided the hyaluronic acid (Ialuset) and the simple emollient, and ensured the monitoring of the trial”. |

Leonardi 2008

| **Methods** | **Design:** Double-blind randomised, vehicle-controlled clinical trial  **Setting:** European institute of Oncology (IEO), Milan, Italy  **Date of study:** June 2004 – May 2005 |
| --- | --- |
| **Participants** | **Inclusion criteria:**  Breast cancer patients scheduled to receive postoperative radiation treatment |
| **Interventions** | **Intervention:**  A: MAS 065D (Sinclaire Pharmaceuticals Ltd, Godalming, UK)  Non-steroidal, water in oil cream with barrier-forming, hydrating and anti-inflammatory properties (hyaluronic acid, shea butter, glycyrrhetinic acid, *vitis Vinifera,* and telmesteine.  **Control intervention:**  B: Emollient cream similar in colour and consistency to intervention cream |
| **Outcomes** | **Primary outcomes of the trial:**  - Maximum degree of radiation dermatitis experienced during RT and the follow-up period.  - Skin reaction was visually assessed and recorded with reference to erythema, desquamation, oedema, moist desquamation and ulceration, using the Skin Toxicity criteria of the National Cancer Institute (NCI).  **Secondary outcomes of the trial:**  - Patient's evaluation of itch, pain, and burning within the radiation field, assessed with a 0-10 cm visual analogue scale (VAS).  - Patients fatigue, the severity of symptoms from the patient's perspective, and compliance with study medication.  **Evaluations time points:**  - baseline, weekly intervals for 28 days of RT, and 3 weeks after completion of RT. |
| **Notes** | Sponsor was the manufacturer of the intervention product |

Lievens 1998

| **Methods** | **Design:** Double Blind, placebo-controlled, randomised controlled trial  **Setting:** Oncology treatment setting, (Belgium)  **Date of study:** Not stated |
| --- | --- |
| **Participants** | **Inclusion criteria:**  Head and Neck cancer patients with confirmed diagnosis of the following:  Oral cavity malignancy, oropharynx malignancy, larynx or hypopharynx  Stages T1-T4  **Exclusion criteria:**  Patients with previous radiation treatment were excluded. |
| **Interventions** | **Intervention:**  A: Sucralfate suspension  Patients instructed to take the suspension six times a day in doses of 1 g with regular intervals. Patients given local anaesthetics or systemic painkillers when needed  **Control intervention:**  B: Placebo oral suspension  Patients instructed to take the suspension six times a day in doses of 1 g with regular intervals. Patients given local anaesthetics or systemic painkillers when needed |
| **Outcomes** | **Primary outcomes of the trial:**  - Subjective intolerance  - Mucositis  - Dysphagia  - Dermatitis  - Nausea  Quote: "All patients were scored clinically once a week throughout the whole treatment according to a numerical scoring system developed in our department" "they were given a calendar to record their drug intake and their daily subjective tolerance and symptoms.  Dermatitis scoring:  0- none  1- slight erythema  2- deep erythema  3- dry desquamation  4- spotted epidermolysis  5- confluent epidermolysis  6- necrosis |
| **Notes** |  |

Liguori 1997

| **Methods** | **Design:** Double-blind- placebo-controlled randomised controlled trial  **Setting:** Department of Radio- Oncology, Ospedale San Giovanni, CH- 6504 Bellinzona, Switzerland  Department of Radio- Oncology, Centre Hospitalier Universitaire Vaudois, Lausanne, Switzerland  (Switzerland)  D**ate of study:** Not stated (20 months of accrual over the two institutions) |
| --- | --- |
| **Participants** | **Inclusion criteria**  Male and Female patients between the ages of 20-85 years of age with either a head and neck, pelvic or breast carcinoma of any stage and given a fractionated radiation therapy.  **Exclusion criteria:**  Patients with previous cutaneous disease or those suffering from any systemic disorder known to delay the skin healing process such as diabetes or severe renal failure. |
| **Interventions** | **Intervention:**  A: Hyaluronic acid  Ialugen , Insititut Biochimique SA (IBSA) Laguna, Switzerland  Adequate instruction given.  Application twice daily No concomitant medication  (Using the flat part of a tongue-depressor, sufficient amount of hyaluronic acid cream or placebo cream (0.4 mg/cm2) was applied to the irradiated skin area twice a day: the first application 1–2 h after the morning radiation treatment session, the second in the evening. The topical treatment of the irradiated area was continued over a 6-  week period whereas the post-radiotherapeutic follow-up lasted four weeks. Patients were instructed to make self application of the cream during the weekends)  **Control intervention:**  B: Placebo  Application twice daily No concomitant medication  (the two formulations  were identical in appearance and could not be distinguished from each other.    The base of the placebo creams was the same and consisted of polyethylenglycol 400 monostearate, lipidic phase, glycerol, 70% sorbitol solution, preservatives, fragrance and purified water.    Institut Biochimique S.A.  (IBSA), Lugano, Switzerland.    Exactly the same instruction were given to the placebo group) |
| **Outcomes** | **Primary outcomes of the trial:**  Skin reaction:  - A scale is used, as list below:  1, light epidermal irritation (consisting of the onset of skin redness, possibly associated to  slight tenderness);  2, erythema with dry desquamation;  3, exudate ,50%;  4, exudate .50%;  5, ulcer.  - This study did not provide any information on validation of the scale  The healing process of an ulcer:  1- minimum and maximum lesion diameters (mm)  2- Lesion cleansing  3- Beginning of the proliferation process and proliferation of the tissue granulation  4- re-epithelialisation  Physician judgement on the global therapeutic efficacy and the tolerability:  0- poor  1- fair  2- good  3- excellent  Any side effects  **Evaluation time points:** A 10 week period follow-up for each patient- during radiation treatment and four weeks post radiation treatment |
| **Notes** | Sponsored by the Institut Biochimique (IBSA), a pharmaceutical company. |

Lin 2006

| **Methods** | **Design:** Double-blind, Placebo controlled, randomised controlled trial  **Setting:** Department of Radiation Oncology, Chi-Mei Foundation Medical centre, Yungkang City, (Tainan, Taiwan)  **Date of study:** January 2003 to August 2004 |
| --- | --- |
| **Participants** | **Inclusion criteria:**  Patients over the age of 18 years with pathologically established head and neck cancers receiving radiation therapy. Patients were receiving radiation to the more than one third of the buccal mucosa and did not have previous radiation treatment for head and neck cancer and did not have diabetes.  **Exclusion criteria:**  Concurrent chemotherapy was not excluded. |
| **Interventions** | **Intervention:**  A: Zinc supplementation  Pro-Z 25 mg, Banner Pharmacaps, High Point, NC.  3 capsules daily from start to the end of treatment.  **Control intervention:**  B: Placebo capsules  3 capsules daily from start to the end of treatment. |
| **Outcomes** | **Primary outcomes of the trial:**  RTOG (skin reactions) and RTOG (Mucosa membrane) |
| **Notes** |  |

Ma 2007

| **Methods** | **Design:** Randomised Controlled Trial  **Setting:** Radiotherapy centre of Oncology department of the second hospital affiliated to Xian Jiaotong University; Tumour Radiotherapy Department of the First affiliated to Xian Jiaotong University, (Xian, China)  **Date of study:** May 2000 to December 2005 |
| --- | --- |
| **Participants** | **Inclusion criteria:**  Patients with Breast ca, nasopharyngeal ca, oesophageal ca, lung ca, Chest, head and neck, armpit and others, receiving radiation treatment |
| **Interventions** | **Trial I:**  **Intervention:**  A:  After each time of radiation treatment Lian Bai Liquid was externally applied to the skin 1cm large than the irradiated area, 3-4 times a day, until the end of the treatment course, and the patient should put on clothes after the liquid was dried.  **Control intervention:**  B:  The patients were given routine nursing after radiation treatment, and asked to expose their irradiated area to avoid friction, wear cotton clothes, not to expose their irradiated area to the sun or wind, not to bathe with very hot water, not to use strongly stimulant detergent, not to scratch but pat the skin if felt itchy.  Trial number 2 (Additional 92 patients, n=92)  **Trial 2:**  **Intervention:**  C:  Grade III skin reaction was cleaned with 0.9% normal saline to remove the exudates, desquamated skin and necrotic tissue, then externally applied with lian bai liquid by an aseptic cotton stick, 3-4 times a day for 2 weeks as a course of treatment.  **Control intervention:**  D:  The affected area with grade III skin reaction was cleaned with 0.9% normal saline and then applied externally with norfloxacin power, 3 times a day for 2 weeks as a course of treatment. |
| **Outcomes** | **Primary outcomes of the trial:**  CTC V2.0 standard stipulated by NCI for classifying radiation induced dermatitis. The clinical effects in all the 4 groups were recorded and evaluated every week. |
| **Notes** | There were two trials in this paper. The patients appeared to be not-overlapping between the trials. However, this was not made explicit in the paper. |

Maiche 1994

| **Methods** | **Design:** Randomised controlled trial  **Setting:** Department of Radiotherapy and oncology of Helsinki  **Date of study:** Not stated |
| --- | --- |
| **Participants** | **Inclusion criteria**:  Women operated on for local breast cancer were included  The patients received 2 Gy per fraction 5 times a week up to a total dose of 50Gy given without split pause. |
| **Interventions** | **Intervention:**  A: Chamomile cream  Kamillosan is a standardised extract of chamomile flower heads.  The drugs were applied gently to the skin twice daily, the first application 30 min before irradiation and the second before bed time throughout the radiation treatment course.  **Control intervention:**  B: almond ointment  Almond ointment is a natural product.  The drugs were applied gently to the skin twice daily, the first application 30 min before irradiation and the second before bed time throughout the radiation treatment course. |
| **Outcomes** | **Primary outcomes of the trial:**  Skin reaction:  0= no change  1= Light erythema  2= Dark erythema  3= moist erythema |
| **Notes** |  |

Mak 2005

| **Methods** | **Design:** Randomised controlled trial  **Setting:** Department of Clinical Oncology, Prince of Wales Hospital, Hong Kong  **Date of study:** Feb 2001- Feb 2003 |
| --- | --- |
| **Participants** | **Inclusion criteria:**  Patients with nasopharyngeal cancer who developed irradiation moist desquamation skin reaction during curative intent radiation therapy  **Exclusion criteria:**  - Patients with previous radiation therapy to the head and neck region  - There was evidence of tumour on the skin  - Patients with clinical wound infection at initial entry into the study  Patients were discontinued from the study if their physical condition deteriorated due to systemic disease. |
| **Interventions** | **Intervention:**  A: Non-adherent absorbent dressing  The non-adherent absorbent dressing was applied on the wound after cleansing by the study nurse on the day of assessment. Patients were taught to apply the dressing themselves on the other days. The dressing was secured with tape at each corner so as to avoid sticking as less area of skin as possible.  **Control intervention:**  B:Gentian Violet  This group received the usual care that comprised a wound care and individual teaching of wound cleansing with homemade salted water. Patients were taught to apply the gentian violet topically on the wound after cleansing by cotton stick. |
| **Outcomes** | **Primary outcomes of the trial:**  **Wound healing**  Wound-healing time was defined as time, in days, between recruitment and observation of complete re-epithelialisation and absence of moist desquamation and burning.  “For patients with multiple wounds, the worst wound would be follow-up along time for consideration in the primary end point assessment, and healing defined as the return of complete skin integrity of all wounds.”    **Secondary outcomes of the trial:**  Wound size  Wound pain score (using 5point- Won/Baker Faces Rating Scale, 0=no pain, 5=very painful)  Presence or otherwise of signs of infection  - erythema and or edema of the surrounding normal tissue  - increased drainage  - change in the nature of drainage from serous to purulent  - increased tenderness in and around the reaction site  - systemic signs of infection, including fever, leukocytosis. A routine wound swab was taking from microbiological growth at the entry of study and also when signs of infection developed.  Mood disturbance (using the Chinese 65 item Profile of Mood State – POMS)  Neck mobility (movement), sleep problem, social isolation, and appearance disturbance were assessed by 4 single questions of 10-point Likert scale, with higher scores indicating more distress.    **Assessment time-points:**  Recruitment day, then once every 2 days thereafter |
| **Notes** |  |

Merchant 2007

| **Methods** | **Design:** Randomised Controlled Trial  **Setting:** Memphis, TN, USA  **Date:** Not stated |
| --- | --- |
| **Participants** | **Inclusion criteria:**  - Age older than 3 years and younger than 21  - a diagnosis that required external-beam irradiation  - No prior history of radiation therapy at the site to be evaluated  - A prescribed total dose of radiation therapy greater than or equal to 23.4 Gy  - No anticipated use of superficial tissue compensators (“’bolus”)  - No pre-existing dermatologic condition that would preclude the evaluation of the skin at the site to be treated  - No contraindications to the use of the study treatments or any of their components  - Adequate performance status as determined by the ECOG scale (0-3) |
| **Interventions** | **Intervention:**  A: APP skin cream  The APP skin cream (Ocular Research of Boston (ORB), Inc, Boston, MA) is a novel oil-in-water emulsion that was prepared in an FDA-approved facility under cGMP guidelines, but it is not commercially available. The active ingredients of APP cream are triglycerides and phospholipids preserved with benzyl alcohol, methyl paraben, propyl paraben, and diaxolipinyl urea. It was applied topically and liberally to the affected area with the bare hand. Application of the cream was accomplished with the ventral surface of the fingers using a rotary motion of the fingers with light pressure to the skin. The cream was massaged into the skin until the surface of the skin no longer felt greasy. Inadequate application was noted by the appearance of a white residual film on the skin.  Control intervention:  B: Aloe Vera Gel  The aloe vera gel which was commercially available, contained water, aloe vera, D-panthenol, triethanolamine, carbomer 934P, hyaluronic acid, potassium sorbate, diazolidinyl urea, methylparaben, and propylparaben. The gel was applied in a manner identical to that described above for the APP cream. |
| **Outcomes** | **Primary outcomes of the trial:**  - Skin care failure which included onset of moderate-to-severe dryness, pruritus, erythema, and dry desquamation  - Subjective skin comfort assessment (rated by patient or parent) (15 items on a 4 point scale)  - Clinical dermatologic assessment  - Assessment by the CTCAE version 1.0 (1- no change, 2-scattered macular or popular eruption or erythema that is asymptomatic, grade 3- scattered macular or papular eruption erythema with pruritis or other associated symptoms; grade 4- generalised symptomatic macular, papular or vesicular eruption; grade 5- exfoliative dermatitis or ulcerating dermatitis.  **Assessment time points:**  Initiation of radiation treatment, weekly during treatment and at the time of first follow-up examination (4-6 weeks after completion of radiation treatment. |
| **Notes** | - This study was supported by a Cancer Centre Support Grant from the National Cancer Institute and the American Lebanese Syrian Associated Charities.  - The phospholipid skin cream was supplied by Ocular Research of Boston (ORB) Inc, Boston, MA. |

Miller 2011

| **Methods** | **Design:** Double-blind, randomised controlled trial  **Setting:** The North Central Cancer Treatment Group  **Date of study:** September 21, 2007, and December 7, 2007 |
| --- | --- |
| **Participants** | **Inclusion criteria:**  - adults (age > 18 years) with histologic proof of a primary invasive breast carcinoma or ductal carcinoma in situ who were to undergo a planned course of continuous, definitive, or adjuvant external beam RT to the whole breast as part of breast conservation therapy or to the chest wall as a part of postmastectomy RT (minimal prescription dose, 50.0 Gy). Treatment of the regional lymph nodes, including the axillary, supraclavicular, and internal mammary lymph nodes, was permitted. The daily treatment dose was 1.75–2.12 Gy. Patients could enter the trial before receiving the third radiation fraction. An Eastern Cooperative Oncology Group performance status of 0, 1, or 2 was required.    **Exclusion criteria:**  - the presence of inflammatory carcinoma of the breast or a known allergy or hypersensitivity to  mometasone and furoate, imidazolidinyl urea, or formaldehyde.  - the use of leukotriene inhibitors or the use of a prescription or over-the-counter medication that contained hydrocortisone or any other cortisone- or corticosteroid-containing preparation.  - had pre-existing loss of skin integrity or previous RT to the area being treated.  - women who were pregnant or breastfeeding and women of child-bearing age who were unwilling to use adequate contraception during the study period.  - patients with bilateral breast carcinoma  - patients receiving partial (<75%) breast treatment. |
| **Interventions** | **Intervention:**  A: 0.1% MMF cream (Dermabase, Paddock Laboratories,  Minneapolis, MN)  **Control intervention:**  B: Placebo  An identical-appearing placebo cream (Dermabase, Paddock Laboratories, Minneapolis, MN)  **Instructions for both interventions:**  Patients were instructed to apply 3 mL of MMF cream or placebo cream lightly once daily to the area under treatment at not less than 4 hours before or after RT until completion of the prescribed RT course. They were instructed to vary the amount of cream on the basis of body habitus and to cover the entire treated area. No other topical agents were allowed to be used in the RT field while the patient was receiving the study medication. |
| **Outcomes** | **Primary outcomes of the trial:**  - Radiation dermatitis determined by the patient’s health care provider with CTCAE version 3.0.  - The maximal grade of CTCAE skin toxicity during treatment  - CTCAE Symptom experience diary  - Quality of life (Skindex-16)  **Assessment time points:**  - baseline and at weekly intervals during their RT by their treatment providers, and two weeks after RT completion |
| **Notes** |  |

Niazi 2012

| **Methods** | **Design:** Randomised controlled trial  **Setting:** Not stated (Canada)  **Date of study:** Feb 2005- Oct 2007 |
| --- | --- |
| **Participants** | **Inclusion criteria**:  All eligible patients were required to have biopsy-proven cancer of the rectum or anal canal. Patients considered candidates for high-dose RT (50 Gy), either as primary treatment or as perioperative  treatment after or before surgical resection, were eligible. All patients were treated with pelvic RT and concurrent chemotherapy. EBRT fields covered the perineal skin of each patient. Patients were treated in the supine position with effective auto bolusing. Nine anal canal cancer patients required custom bolus, 5 in SCND, and 4 in the standard arm. |
| **Interventions** | **Intervention:**  A: Silver Clear Nylon Dressing (SCND)  SCND wore the dressing, supplied by the manufacturer free of charge, from Day 1 of RT, 24 hours per day 7 days per week, except during RT delivery time. This was continued until 2 weeks after the treatment was completed to take advantage of SCND’s prophylaxis and  intervention qualities.  **Control intervention:**  B: standard skin care  According to the institutional standard, all patients in the control arm used sulfadiazine cream at the time of development of grade 1 skin dermatitis. Patients were advised to wash the cream off at least 4 h prior to RT. |
| **Outcomes** | **Primary outcome of the trial:**  Skin toxicity (CTCAE version 4), this was rated by 10 observers using digital photos. The average score was used by simply summing the ten scores and then divided by 10. ICC among observers were 0.87-0.94. |
| **Notes** |  |

Olsen 2001

| **Methods** | **Design:** Randomised controlled trial  **Setting:** Radiation therapy outpatient clinic in an cancer centre Sylvester Comprehensive Cancer Centre of University of Miami, (USA)  **Date of study:** Not stated |
| --- | --- |
| **Participants** | **Inclusion criteria:**  All patients being treated with radiation therapy who are expected to develop skin reactions. Cancer sites included head/neck, chest, abdomen/pelvis and extremities  **Exclusion criteria:**  Patients receiving radiation to the brain or for gynaecologic cancers where excluded. |
| **Interventions** | **Intervention:**  A: Aloe Vera and soap  Aloe Vera gel: Fruit of the Earth, Irving, TX + Dove soap  Adequate instructions given  Start with first day of radiation, directed to gently cleanse irradiated area with mild unscented soap(provided) Protect from trauma, band aids, tape, prolonged exposure to sunlight and do not rub area. Pat dry and ear loose fitting clothes.  Apply gel to liberally following radiation and encouraged to reapply throughout the day. Average 6-8 times. Gently rinse off gel before treatment.  **Control intervention:**  B: Dove soap  Adequate instructions given  Start with first day of radiation, directed to gently cleanse irradiated area with mild unscented soap(provided) Protect from trauma, band aids, tape, prolonged exposure to sunlight and do not rub area. Pat dry and ear loose fitting clothes |
| **Outcomes** | **Primary outcome of the trial:**  Skin reaction:  Nurses and physician weekly skin scoring using RTOG  Assessments were conducted prior to the first radiation treatment, and weekly during regular scheduled on treatment visits with the physician and nurse. |
| **Notes** | **Comment:** No other potential threats to validity were identified |

Omidvari 2007

| **Methods** | **Design:** Double blind, Randomised controlled trial  **Setting:** Radiation oncology department, Nemazee Hospital, Shiraz Universirty of Medical Science, (Shiraz, Iran)  **Date of study:** August 2005 to October 2005 |
| --- | --- |
| **Participants** | **Inclusion:**  Female patients who underwent modified radical mastectomy for stage II or III pathologically proved breast cancer and in addition to surgery and chemotherapy, needed RT. Between 20-70 years of age, no history of previous RT, Diabetes or systemic connective tissue disorder.  Site: chest wall  **Exclusion criteria:**  Patients receiving concurrent chemotherapy and radiation or were on systemic corticosteroids were excluded. |
| **Interventions** | **Intervention:**  A: Bethamethasone  Bethamethasone 0.1 %  - Verbal instructions on application of drug at the beginning of randomisation and then during weekly observations. Apply two times a day from the first day of RT until 2 weeks after the completion of RT. Use the same volume of formulation on each application and clean the site before each radiation  B: Petrolatum  Petrolatum (only used for its emollient effect)  - Verbal instructions on application of drug at the beginning of randomisation and then during weekly observations. Apply two times a day from the first day of RT until 2 weeks after the completion of RT. Use the same volume of formulation on each application and clean the site before each radiation.  **Control intervention:**  C: No treatment  No instructions  (unclear if these participants used any type of cream independent of the study) |
| **Outcomes** | **Primary outcome of the trial:**  Skin reaction: RTOG |
| **Notes** |  |

Paterson 2012

| **Methods** | **Design:** Randomised intra-patient controlled trial  **Setting:** Dunedin Regional Hospital (DUN), WellingtonRegional Hospital(WGN), Palmerston North Regional Hospital (PLN) and AucklandRadiation Oncology (ARO), New Zealand  **Date of study:** Date: March 2011-May 2012 |
| --- | --- |
| **Participants** | **Inclusion criteria:**  All women receiving radiation therapy for breast cancer at Dunedin Regional Hospital (DUN), Wellington Regional Hospital (WGN), Palmerston North Regional Hospital (PLN) and Auckland Radiation Oncology (ARO) who had a mastectomy were screened for recruitment. Participants also had to be able to return to the department for weekly assessments after the completion of treatment.  **Exclusion criteria:**  Previous radiation therapy to the ipsilateral chest wall, metastatic disease, breast reconstruction, impaired mobility and a Karnofski performance status score of less than 70. |
| **Interventions** | **Intervention:**  A: Mepilex Lite Dressings  Mepilex Lite dressings were positioned by the research radiation therapist on half of the area where erythema was present; the other half continued to be treated with aqueous cream (control). Distribution of the dressings was randomised and their localization on the chest wall was indicated by a semi-permanent marker pen, so that patients could accurately reposition the dressings after showering. A tracing of the area was also taken in case the marks washed off and to facilitate dose estimations using the RT planning system.  **Control intervention:**  B: Aqeuous cream  Aqueous cream was applied twice a day to the control patches and other parts of the chest wall that were not part of the study area. Hydrocortisone cream (1%) was prescribed by some centres to reduce excessive itching when appropriate. The surface area of the skin patches analysed ranged from 50 to 220 cm^2^. |
| **Outcomes** | **Primary outcomes of the trial:**  - Severity of skin reactions (Radiation induced skin reaction assessment scales developed by Noble-Adams, and modified by MacBride. The research radiation therapist scores the visible extent of the skin reactions (with separate scores for erythema, dry desquamation, moist desquamation and necrosis). The patient scores the level of pain, itchiness, burning and effect on day to day life. The researcher and patient scores are then summed to give the combined RISRAS score    **Assessment time points:** RISRAS scores were determined three times a week (on Mondays, Wednesdays and Fridays) from the moment erythema was visible until completion of radiation treatment. Weekly assessments were done after completion of treatment until all acute skin reactions had cleared up (usually four to five weeks after completion of treatment). All RISRAS scores for each area were added up and divided by the number of assessments, yielding an average RISRAS score for that area. |
| **Notes** | Patients were serving as their own control. There may be potential unit of analysis issues |

Pommier 2004

| **Methods** | **Design:** Randomised controlled trial  **Setting:** Department of Radiation Oncology, Centre Leon Berard, (Lyon, France)  **Date of study:** October 1999 to June 2001 |
| --- | --- |
| **Participants** | **Inclusion criteria:**  Women referred to the department of radiation treatment, 18 -75 years of age, Non metastatic adenocarcinoma, post either lumpectomy or mastectomy, with or without adjuvant postoperative chemotherapy or hormonal treatment.  Cancer sites included Breast/chest wall and if relevant sub mammary fold, armpit, internal mammary nodes and supra clavicular nodes  **Exclusion criteria:**  No concomitant chemotherapy was allowed.  Women with bilateral or in situ breast cancer, patients who were allergic to either of the two agents, and pregnant women were excluded. |
| **Interventions** | **Interventions:**  A: Calendula  Calendula, Boiron Ltd, Levallois-Perret, France  Patients were asked to start topical application of their ointment on the irradiated skin at the onset of radiation treatment, twice a day or more depending on the occurrence of dermatitis and pain until the completion of the radiation therapy. No other prophylactic creams, gels or lotions were allowed, however physicians were free to treat established dermatitis of grade 2 or higher and/or allergy as they considered appropriate.  **Control intervention:**  B: Trolamine  Trolamine, Biafine; Genmedix Ltd, France  Patients were asked to start topical application of their ointment on the irradiated skin at the onset of radiation treatment, twice a day or more depending on the occurrence of dermatitis and pain until the completion of the radiation therapy. No other prophylactic creams, gels or lotions were allowed, however physicians were free to treat established dermatitis of grade 2 or higher and/or allergy as they considered appropriate. |
| **Outcomes** | **Primary outcomes of the trial:**  Skin reactions: RTOG  **Secondary outcomes of the trial:**  - Pain (0-10cm VAS)  - Treatment interruption  - Patient satisfaction  - Quantitiy of the agent used |

Primavera 2006

| **Methods** | **Design:** Double blind, vehicle controlled randomised controlled trial  **Setting:** San Gallicano Dermatological Insititute, (Rome, Italy)  **Date of study:** May 2003, May 2004 |
| --- | --- |
| **Participants** | **Inclusion criteria:**  Patients were eligible for the study if they were being treated with radiation treatment  for breast cancer, were 18 years or over, and had signed an Institutional Review  Board (IRB) – approved informed consent form. Patients received a total of 50–70 Gy over a period of 4–6 weeks in daily fractions Monday to Friday (linear accelerator VARIAN CLINAC L 2100). The use of other topical products on test areas was prohibited throughout the study period.    **Exclusion criteria:**  Patents with history of previous radiation treatment to the area, concomitant chemotherapy, and other skin conditions that might affect the outcome of the study. Patients with known hypersensitivity or previous allergic reaction to any of the components of MAS065D, or the vehicle cream, as well as pregnant or nursing women, were also excluded. |
| **Interventions** | **Intervention:**  A: MAS065D- (XCLAIR TM)- a Hyaluronic acid-based formulation  (key ingredient: Hyaluronic acid, glycyyhrtinic acid and shea butter)  Patients were instructed to apply the study substance three times daily, or more if required, throughout the duration of radiation treatment and for two weeks afterwards.  **Control intervention:**  B: The vehicle was an emollient base cream, similar to MAS065D but without the key ingredients.  *Patients act as their own control. Patients were randomised into two groups: one received MAS065D on section 1 and vehicle on section 2, and the other group the reverse. |
| **Outcomes** | National Cancer Institute skin radiation toxicity criteria (grade 0–4) (17); erythema rating (18), using the MexameterTM MX16 (Courage and Khazaka electronic GmbH Koln, Germany); transepidermal water loss (TEWL), using the TewameterTM (Courage and Khazhaka, electronic GmbH Koln, Germany); skin hydration, using the CorneometerTM CM 825 (Courage and Khazhaka, electronic GmbH Koln, Germany).  Patient’s view of itch and pain on a Visual Numerical Scale (0–10); and adverse events.  At the final visit both the Investigator and the patients were asked for their preference between the two creams. |
| **Notes** | - This publication was supported by a grant from Sinclair Pharmaceuticals  - Same group of patient, acting as self-controlled, unit of analysis issues.  - This was a preliminary study, therefore the sample size was chosen on empirical basis. Twenty patients were expected by the trial authors to be sufficient to gain an initial impression of the differences between the two groups and to provide data needed for the planning of future studies. |

Ribet 2008

| **Methods** | **Design:** Randomised controlled trial  **Setting:** Seven French centres of radiation, (France)  **Date of study:** Not stated |
| --- | --- |
| **Participants** | **Inclusion criteria:**  Patients who had breast cancer or ENT cancer requiring treatment photon or electron therapy for 6 weeks at 5weekly sessions each 2Gy on a surface of intact skin between 30 and 400 cm2. Adult women included in the study should be contraceptives efficient started over a month before inclusion.    **Exclusion criteria:**  Any skin disease and / or chemotherapy may interfere with the clinical signs observed, radiation treatment received within three weeks prior to the study the area to be irradiated, pregnancy, breastfeeding or allergy known to one of the components of the topical tested. |
| **Interventions** | **Intervention:**  A: The gel ETA  The ETA gel is formulated with 99% Avene Thermal Spring Water and 1% gel agent classically used in dermatology. The gel was packed in white tubes of 10 ml. They were applied 5 times per day for 10 weeks (6 weeks of treatment radiation treatment and four weeks later).  **Control intervention:**  B: The base cream trolamine.  Base cream trolamine, Biafine® (Laboratoire Medix), packaged in white tube of 93 g.  They were applied  5 times per day for 10  weeks (6 weeks of treatment radiation treatment and four weeks later). |
| **Outcomes** | **Primary outcomes of the trial:**  Time to first sign of dermatitis rated by patients: Erythema, edema, scaling, and lesions oozing, 0- absent, 1= mild, 2- moderate, 3= severe) Global score: (range from 0-12)  Radiodermatitis: NCI grade 0- no symptoms, grade (mild erythema or dry desquamation, grade 2- moderate to severe erythema, wet  desquamation injury, less than 1.5 cm moderate edema, grade 3- moist desquamation lesions by converging over 1.5cm with severe edema, grade 4- necrotic skin, ulcerate with hemorrhagic lesions.  **Secondary outcomes of the trial:**  - Median time to onset of pruritus and pain assessed daily by the patient according to a rating from 0 to 3.  - Overall efficacy was judged by the investigator according to the following scale: 0- poor, 1- poor, 3- good, 4- excellent |
| **Notes** | All authors appeared to be affiliated or employees/ owner of the topical intervention developer. |

Rizza 2010

| **Methods** | **Design:** Randomised controlled trial  **Setting:** Department of Radiotherapy at Humanitas Centro Catanese di Oncologia, (Catania, Italy)  **Date of study:** Not stated |
| --- | --- |
| **Participants** | **Inclusion criteria:**  Volunteer women who were undergoing planned course of RT after breast conserving surgery referred to the Department of Radiotherapy.  Age, tumour stage, smoking status, skin type and breast size were recorded.  **Exclusion criteria:**  Patients receiving concomitant systemic anti-cancer treatment or prior RT, exhibiting cutaneous disease were excluded. |
| **Interventions** | **Interventions:**  A: Formulation A  3% w/w blend of natural extracts containing *capprais spinosa, opuntia coccinellifera and Olive leaf extracts*  - Instructed to apply a thin layer of cream twice daily to the treatment area form the first day of radiation treatment. The formulation was not applied within 4 hours of radiation.  B: Formulation B  Biafian, Isttituto Ganassini, Italy.  - Instructed to apply a thin layer of cream twice daily to the treatment area form the first day of radiation treatment. The formulation was not applied within 4 hours of radiation.  **Control intervention:**  C: Untreated |
| **Outcomes** | **Primary outcomes of the trial:**  - The Modified RTOG and Erythemic Index (EI) |
| **Notes** |  |

Roy 2001

| **Methods** | **Design:** Randomised controlled trial  **Setting:** Department of Radiation oncology, centre hospitalier Universitaire de Quebec, Canada  **Date of study:** between April 1998 and June 1998 |
| --- | --- |
| **Participants** | **Inclusion criteria:**  (a), patients scheduled to be treated with adjuvant external beam radiation therapy of the breast or chest wall; (b), a total prescribed dose superior to 40 Gy; (c), the use of megavoltage X-rays; and (d), written informed consent.  **Exclusion criteria**:  (a), patients less then 18 years old; (b), patients treated with electron beam therapy (except for local boost); (c), patients in whom adherence to the washing/not washing instructions could be compromised  (for example confused patients); (d), patients in whom an adequate follow-up was not achievable, such as patients from satellite centres that were not be seen in follow-up at the radiation oncology department due to distance |
| **Interventions** | **Intervention:**  A: No washing  **Control intervention:**  B: Washing with Soap  **Other instructions:** These following general recommendations were given to patients in both groups: do not erase ink marks; do not apply deodorant, lotion, cream, make up, perfume or any other product on the irradiated skin unless prescribed by your physician; do not apply water soaks to relieve itching or pain; avoid the use of dressing on the treatment field; and avoid exposure to the sun. |
| **Outcomes** | **Primary outcomes of the trial:**  French translation of the Radiation Therapy Oncology  Group (RTOG) acute toxicity scale: grade 0, no change over baseline; grade 1, follicular, faint or dull erythema, epilation, dry desquamation and/or decreased sweating;  grade 2, tender or bright erythema, patchy moist desquamation and/or moderate edema; grade 3, confluent, moist desquamation other than skin folds, pitting edema; grade 4, ulceration, haemorrhage, necrosis  - Mean time to maximal toxicity  - Maximum toxicity scores at any time Grade 0 |
| **Notes** | - They conducted a sample size calculation requiring 55 patients per arm. However, they were unable to reach the number, and made a priori decision to only reach 50 patients.  - At four weeks after treatment completion, only 38 participants completed data collection in the no washing group, and 32 in the washing group. |

Schmuth 2002

| **Methods** | **Design:** RCT  **Setting:** Department of Radiation Therapy, Innsbruck  **Date of study:** April 1999 January 2000 |
| --- | --- |
| **Participants** | **Inclusion:** Eligible patients were women aged 18-80 diagnosed with breast cancer and receiving RT following breast conservation surgery, at the Department of Radiation Therapy, Innsbruck. |
| **Interventions** | **Intervention:**  A: dexpanthenol  0.5% dexpanthenol  Trade name: Bepanthen,  Hoffmann, LaRouche, Berne, Switzerland.  - Patients were instructed to apply cream to irradiated field twice a daily from the initiation of the RT.  - Patients were instructed not to use any other topical medications, emollients or powders during this period.  Adequate instructions  **Control intervention:**  B: Methylsprednisolone  0.1% Methylsprednisolone  Tradename: Advantan, Schering, Vienna, Austria.  - Patients were instructed to apply cream to irradiated field twice a daily from the initiation of the RT.  - Patients were instructed not to use any other topical medications, emollients or powders during this period.  Adequate instructions |
| **Outcomes** | **Primary outcomes of the trial:**  - Level of skin toxicity at peak: ‘clinical course’ (limited description provided), Scale: 0-none, 1-mild, 2-moderate, 3-severe.  - General health outcome(SF-36)  and Skin related health outcomes (Skindex). |
| **Notes** |  |

Shell 1986

| **Methods** | **Design:** Randomised controlled trial  **Setting:** Not stated, (USA)  **Date of study:** Not stated |
| --- | --- |
| **Participants** | **Inclusion criteria:**  All patients receiving radiation therapy to either head and neck, chest or back, with a resulting moderate or severe radiodermatitis. |
| **Interventions** | **Intervention:**  A: MVP dressing  MVP dressings were applied without tension to the reaction site so that the dressing covered a 2 inch margin of less affected or normal skin at the periphery.  Dressing was changed daily to once every 3-4 days  **Control intervention:**  B: Hydrous lanolin dressings  Hydrous lanolin dressings were spreading a thick layer of hydrous lanolin on sterile gauze dressings. The lanolin gauze was applied directly to the radiation site, wrapped with roll gauze and secured with tape on the gauze rather than on the patient skin./ Dressing was changed once or twice a day |
| **Outcomes** | **Primary outcomes of the trial:**  RISRs were graded into moderately severe reactions (dry desquamation and brisk erythema  Severe reactions- vesicle formation and moist desquamation with areas of partial and full thickness skin loss and pronounced erythema.  Pain- 0-5, 0 representing no pain, and 5 representing excruciating pain |
| **Notes** | The study was reduced from 21 to 16 patients after they have developed wound complications.  This study was too small to detect any differences |

Théberge 2009

| **Methods** | **Design:** Randomised controlled trial  **Setting:** RT department at the Hotel-Dieu de Quebec, Canada  **Date of study:** Not stated |
| --- | --- |
| **Participants** | **Inclusion criteria:**  - All patients with breast cancer who were scheduled to undergo RT at the cancer centre were potentially eligible for randomisation, if they were  Inclusion:  - >18 years old  - Had histologically proven breast cancer  - Were receiving adjuvant external beam RT to the breast or chest wall (with or without lymph nodes associated  - Were prescribed a dose of >40 Gy  **Exclusion criteria:**  Treatment with electron beam therapy (except for local boost), concomitant chemotherapy, partial breast RT or breast brachytherapy, previous ipsilateral breast or chest wall RT, previous Chemotherapy for another neoplasia, pregnancy or lactation, and a known allergy or hypersensitivity to deodorant. |
| **Interventions** | **Intervention:**  A: Deodorant Group  Patients were instructed to use the product on a daily basis during RT. Only deodorant without aluminium was permitted. Antiperspirant containing aluminium was forbidden. It is the only criterion used to exclude a deodorant.  Patients received a list of deodorants to help guide their choice and the deodorant had to be approved by the research nurse. (until the treatment had ended)  **Control intervention:**  B: No Deodorant. |
| **Outcomes** | **Primary outcomes of the trial:**  - RTOG for skin toxicity  - EORTCQoL-C30 Scale  - CTCAE for pain and purities  - Inhouse scale for sweating |
| **Notes** | - No sample size calculation was conducted/  - Some participants in the both groups were excluded from the analysis. The authors did not account why they were lost to follow-up.  - Authors did not report on whether participants purchased and used any deodorant during the treatment. They did not report on compliance. |

Watson 2012

| **Methods** | **Design:** Randomised controlled trial  **Setting:** Tom Baker Cancer Centre, Calgary, Canada  **Date of study:** Dec 2008- July 2010 |
| --- | --- |
| **Participants** | **Inclusion criteria:**  Grade 0, I, and II breast cancer and scheduled to receive EBRT to the breast at the Tom Baker Cancer Center  **Exclusion criteria:**  Patients were excluded if they had undergone total mastectomy, had nodal involvement requiring  axillary RT, or had a previous known sensitivity or allergy to antiperspirants. |
| **Interventions** | **Intervention:**  A: Antiperspirant  The experimental group received the same standard skin care teaching but was instructed to use a consistent study-supplied antiperspirant once daily with two up-down strokes for the duration of treatment and follow-up. For the purposes of our study, we selected an antiperspirant that contained a moderate aluminium content (21%).  **Control intervention:**  B: No antiperspirant  The control group was instructed to follow the existing institutional standard skin care protocol, which included instructions that no antiperspirant or deodorant was to be used during treatment or until the skin had returned to normal after treatment (usually 2-3 weeks). |
| **Outcomes** | **Primary outcomes of the trial:**  National Cancer Institute Common Toxicity Criteria for Adverse  Events, version 3, toxicity grading criteria  **Assessment time points for primary outcomes:**  Assessment Timepoints: Weekly and 2 weeks post treatment  **Secondary outcomes of the trial:**  Quality of life: The Functional Assessment for Chronic Illness Therapy’s questionnaire for the breast population (FACIT-B, version 4)  Body odour: A locally designed additional subset of questions specific to body odour and the sense of well-being associated with using or not using antiperspirant was added.  **Assessment time points for secondary outcomes:**  Day 1, last day of treatment and 2 weeks post treatment. |
| **Notes** |  |

Wells 2004

| **Methods** | **Design**: Randomised controlled trial  **Setting**: Western General Hospital Edinburgh and Ninewells Hospital, (Dundee, UK)  **Date of study**: November 2000 – October 2002 |
| --- | --- |
| **Participants** | **Inclusion criteria:**  - New patients attending radiation treatment at Western General and Ninewells Hospitalsprescribed dose of > 40 Gy.  - Patients with Breast or Anorectal or Head & Neck cancers  -Stage of disease: Not Stated  -Breast or Anorectal or Head and neck.  -Hospital setting |
| **Interventions** | **Intervention:**  A: Aqeuous cream  **Control intervention:**  B: Sucralfate Cream  **Instructions:**  All patients given identical advise re: washing the treatment area and an unperfumed soap.  Patients were advised to apply a thin layer of cream to the treatment area  from the first day of the treatment  The cream manufactured buy Tayside pharmaceuticals  Adequate instructions: simple instructions for a basic task |
| **Outcomes** | **Primary outcomes of the trial:**  - Dermatology Life Quality Index (DLQI)  - Modified RTOG scale  0-None, 1- Follicular, faint or dull erythema, 1.5- Dry desquamation, 2-Tender or Bright Erythema, 2.5- Patchy moist desquamation, 4- Ulceration, Haemorrhage, necrosis. |
| **Notes** |  |

Wheat 2006

| **Methods** | **Design:** Randomised controlled trial  **Setting:** Not stated (Australia)  **Date of study:** Not stated |
| --- | --- |
| **Participants** | **Inclusion criteria:**  Histologically confirmed breast ca  >50 Gy to primary site  >18 yo  Zubrod performance status of 0 or 1  Ability conform to treatment regime  **Exclusion criteria:**  Concurrent chemotherapy  Rash, ulceration, haemorrhage or unhealed scar in treatment area  Allergy to wheatgrass extract  Local involvement of cancer in the skin  History of connect tissue disorder  Prior radiation treatment to treatment area |
| **Interventions** | **Intervention:**  A: Wheatgrass extract  **Control intervention:**  B: Sobolene |
| **Outcomes** | **Primary outcomes of the trial:**  - Time taken to peak time  - QoL at 5 weeks and 6 weeks  ONS  ONS radiation skin reaction scoring system (Porock 1999a)  0-     No change  1-     faint or dull erythema, follicular reaction  2-     bright erythema  3-     Dry desquamation with or without erythema  4-     Small to moderate wet desquamation  5-     Confluent moist desquamation  6-     Ulceration, haemorrhage or necrosis |
| **Notes** | This is a study reported twice in two journal articles (see Wheat 2007) |

Wheat 2007

| **Methods** | **Design:** Randomised controlled trial  **Setting:** Not stated, Australia  **Date of study:** Not stated |
| --- | --- |
| **Participants** | **Inclusion:**  20 lumpectomy patients receiving breast radiation. No more information was provided by the trial authors. |
| **Interventions** | **Intervention:**  A: Sorbolene cream  **Control intervention:**  B: Wheatgrass extract  Instructions: The cream was applied three times daily beginning with the commencement of radiation treatment for the duration of the patient’s radiation treatment |
| **Outcomes** | **Primary outcomes of the trial:**  - Peak ONS rating  - Time to peak ONS rating  ONS radiation skin reaction scoring system (Porock 1999a)  0-     No change  1-     faint or dull erythema, follicular reaction  2-     bright erythema  3-     Dry desquamation with or without erythema  4-     Small to moderate wet desquamation  5-     Confluent moist desquamation  6-     Ulceration, haemorrhage or necrosis |
| **Notes** | This study reported some p values and stated that QoL improved for week 2, 3, 5 and 6.  Only week six had a p value <0.05.  However, there are no numerical results, and the text did not state which group had improved QoL. |

Williams 1996

| **Methods** | **Design:** Randomised controlled trial  **Setting:** No details about the cancer centres. A collaborative trial of North Central Cancer Treatment Group and Mayo Clinic, (USA)  **Date of study:**  15 Nov 1990- 30 Dec 1991 (n=194)  16 Dec 1992 to 23 Aug 1993 (n=108) |
| --- | --- |
| **Participants** | **Inclusion criteria**  - Breast cancer patients  - Must have a diagnosis of breast cancer, must have had a planned course of radiation therapy to the breast and/or chest wall involving a minimum field of 10x10 cm and a minimum dose of 50 cGy (including boost).  - Must not have had skin rashes, ulcerations, or poorly healed scars in their radiation ports. |
| **Interventions** | **Stage 1:**  Intervention  A: Aloe Vera Gel  - The gel was to be applied lightly to the treatment field twice a day starting within 3 days of radiation initiation (ideally on the first day).  - Patients were to follow usual skin care precautions given at the start of radiation treatment, such as not applying soap directly to the skin etc.  - No other prophylactic creams/lotions/gels were to be topically applied to the radiation field while a patient was participating in the study.  - However, treatment of established radiation-induced dermatitis was allowed. Patients with pruritus and/ or marked erythema were to use a 1% hydrocortisone cream two to three times daily (different times throughout the day- 1 hour interval min).  If moist desquamation occurs, Domeobro’s soaks were to be used 3-4 times/day  Control intervention:  B: Placebo Gel (inert gel)  **Stage 2:**  Intervention  A: Aloe Vera Gel  B: No gel |
| **Outcomes** | **Primary outcomes of the trial:**  Maximum RISR scores (Yes/ No)  No RISR (by patients and a clinician)  Mild RISR (by patients and a clinician)  Moderate RISR (by patients and a clinician)  Severe RISR (by patients and a clinician)  The chest wall was examined by the physician or oncology nurse (Normal, 1- mild erythema, 2 marked erythema with or without desquamation, 3- moist desquamation and/or ulceration.) |
| **Notes** | -  This study measured maximum scores, but not at particular time point. |

Zhang 2011

| **Methods** | **Design:** Randomised Controlled Trial  **Setting:** Department of oncology, The First Affilicated Hospital of Xinjiang Medical University (China)  **Date of study:** Oct 2008 to Oct 2009 |
| --- | --- |
| **Participants** | **Inclusion criteria:**  head and neck carcinoma patients receiving radiation treatment |
| **Interventions** | **Intervention:**  A: Trolamine (Biafine)  Control intervention:  B: Qingdiyou medication  If grade II and grade III skin reaction appears, these interventions were continued with additional antibiotic |
| **Outcomes** | **Primary outcomes of the trial:**  Chinese translation of the RTOG EORTC  **Assessment time points:**  At the start of treatment, daily during treatment, and “3-6 months” after treatment |
| **Notes** |  |
